# Cucurbiturils mimicked by low polarizability solvents with pre-formed cavities: an empirical model to predict hydrocarbon selectivity†

**DOI:** 10.1039/d1sc06728a

**Published:** 2022-03-21

**Authors:** Md Nazimuddin, Héctor Barbero, Ramin Rabbani, Eric Masson

**Affiliations:** Department of Chemistry and Biochemistry, Ohio University Athens Ohio 45701 USA masson@ohio.edu; GIR MIOMeT, IU CINQUIMA/Química Inorgánica, Facultad de Ciencias, Universidad de Valladolid Valladolid E47011 Spain

## Abstract

Relative binding affinities of a series of nine rigid hydrocarbons towards the cavity formed by a portion of the inner wall of cucurbit[8]uril (CB[8]) and a positive auxiliary guest were determined by competitive ^19^F NMR titrations in deuterium oxide. The corresponding free binding energies were corrected by the hydrocarbon computed solvation energies to obtain their free energies of transfer from the gas phase to the CB[8]/auxiliary guest cavity. These energies correlate linearly with the hydrocarbon static polarizabilities, thereby suggesting that the selectivity is driven, perhaps exclusively, by dispersive interactions between the hydrocarbons and the tailor-made cavity, regardless of the degree of unsaturation of the guests. The free energies of transfer also correlate linearly with the energy released upon introduction of the hydrocarbon into a pre-formed cavity extruded from a solvent (benzene) selected to mimic the polarity and polarizability of the CB[8]/auxiliary probe cavity – and this, with a unity slope. Among other features, this empirical model also accurately predicts the relative binding affinities of various rigid hydrocarbons to CB[6] and CB[7], as well as noble gases to CB[5], when the macrocycles are mimicked with pre-formed cavities in perfluorohexane or perfluorohexane/benzene mixtures, both being notoriously non-polar and non-polarizable environments.

## Abstract 2

Mimicking cucurbiturils with low polarizability solvents and pre-formed cavities allows the *in silico* prediction of their selectivities towards hydrocarbons and noble gases in aqueous solution.

## Graphical abstract

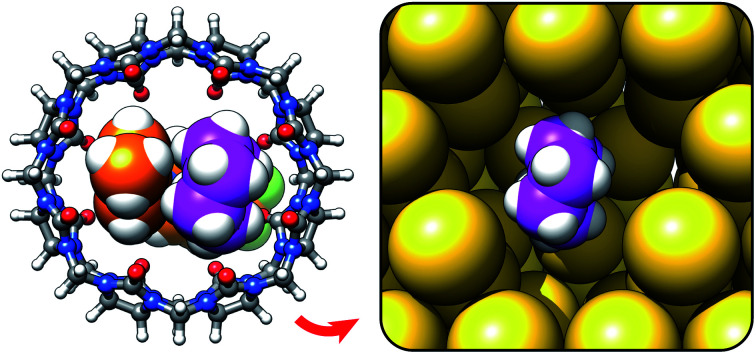

## Introduction

Cucurbit[*n*]urils (CB[*n*]), a family of hollow, pumpkin-shaped macrocycles,^1–5^ encapsulate positively charged, amphiphilic guests in their cavity with extreme affinity in aqueous medium (up to 7 × 10^17^ M^−1^).^6^ As the main driving force for the encapsulation is the ejection of water from the cavity to the bulk, CB[*n*]s can also encapsulate neutral guests like hydrocarbons,^7–12^ with micro-to nanomolar affinities (1.3 × 10^6^, 2.2 × 10^9^ and 1.5 × 10^7^ M^−1^ for cyclopentane, adamantane and diamantane in CB[6], CB[7] and CB[8], respectively).^7,9,10^ While CB[8] does encapsulate hydrocarbons,^9^ it also has the unique ability to form heteroternary complexes with both a hydrocarbon and an auxiliary guest.^11,12^ The CB[8]/auxiliary guest combination thus allows the creation of tailor-made cavities with unique and tunable recognition properties.

**Fig. 1:**
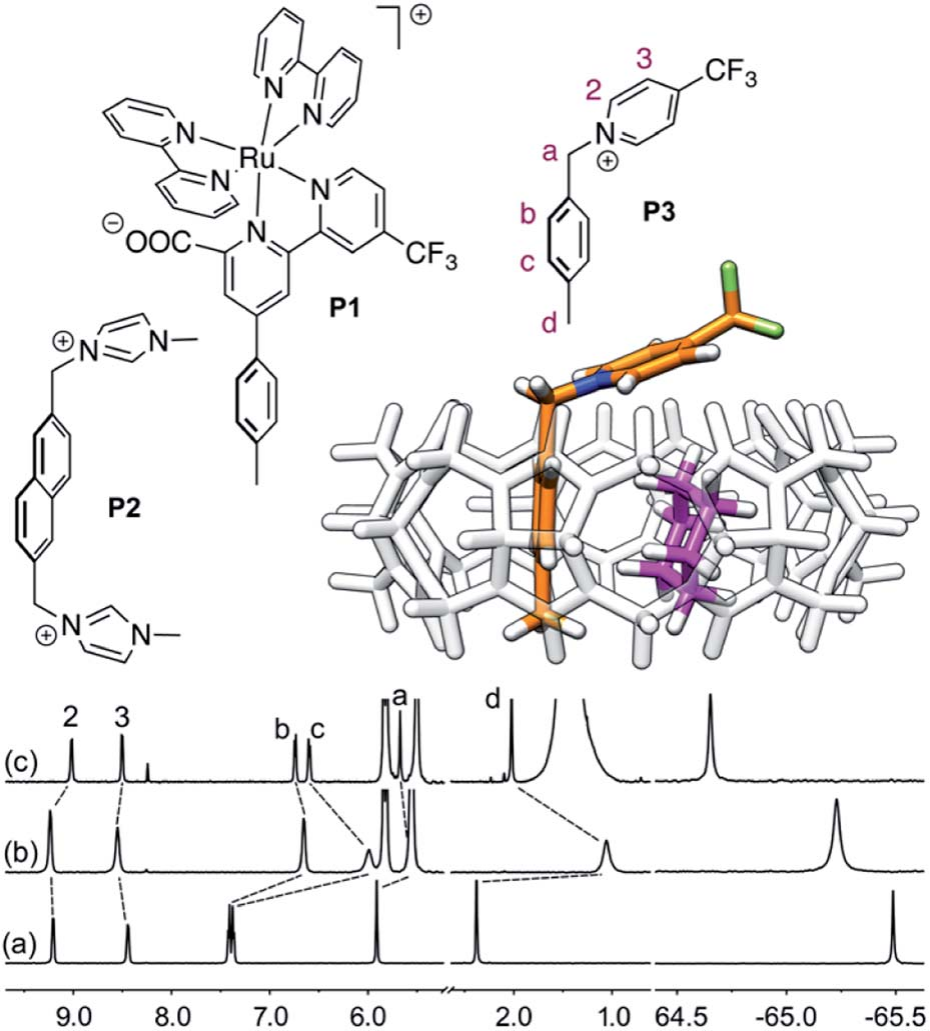
Structures of guests P1,^11^P2 ^12^ and P3. Ternary complex CB[8]·P3·cyclohexane optimized with the semi-empirical method GFN2-xTB^14–16^ in conjunction with the ALPB solvation model.^17^^1^H (left) and ^19^F (right) NMR spectra of (a) guest P3, (b) homoternary complex CB[8]·P3_2_, and (c) heteroternary complex CB[8]·P3·cyclohexane. Chemical shifts in ppm.

In 2017, we showed that saturated hydrocarbons bind the CB[8]/auxiliary probe P1 assembly (see Fig. 1) better than unsaturated ones, and we attributed this selectivity to CH–π interactions between the saturated hydrocarbons and the tolyl unit of probe P1 as being more favorable than “π–π” interactions with unsaturated hydrocarbons.^11^ Two years later however, Nau and Scherman came to the opposite conclusion with assembly CB[8]·P2 (see Fig. 1) – we note here that “π–π” interactions are dispersive in nature, and do not involve p orbital overlap between small aromatic units.^13^ In any case, both studies have their own limitations: 1,3-cyclohexadiene, 1,4-cyclohexadiene, cyclohexene and cyclohexane certainly bind 2, 4, 14 and 160 times better than benzene to the CB[8]·P1 assembly, respectively (see Table 1); however the guest sample size is small. Similarly, Nau and Scherman show that isobutene binds assembly CB[8]·P2 twice better than isobutane, cyclopentene 4 times better than cyclopentane, and benzene and 1,3-cyclohexadiene 3 and 8 times better than cyclohexane, respectively (see Table 1). The small sample size and the mild differences in binding affinities are equally problematic. One could also argue that probe P1 is a large coordination complex and the impact of the Ru tris-bipyridine unit on the binding affinities is unknown. Similarly, probe P2 might adopt multiple, hydrocarbon-dependent conformations (for example, the imidazolium units might not always “cap” the CB[8] portals and interact with the hydrocarbon). Considering these limitations, our goal was to design a much simpler mimic of guest P1 and to test again the impact of hydrocarbon unsaturations on binding affinities to the new CB[8]/probe P3 assembly. The observed trends exceeded expectations, and allow us to propose here a new (and possibly controversial) model to predict relative binding affinities of hydrocarbons to CB[6], CB[7], and CB[8]/auxiliary probe assemblies. We will also show that the validy of this model extends to noble gases binding to CB[5].

**Table 1:** Relative binding affinities of hydrocarbons to assemblies CB[8]·P1 and CB[8]·P3, and their corresponding free energies of transfer from solution and from the gas phase to the cavities of the binary complexes

Hydrocarbon	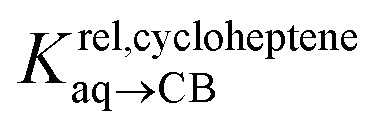 a		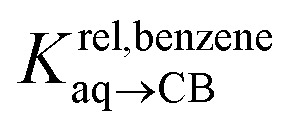 b			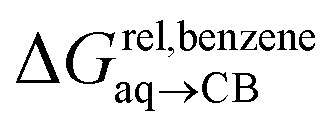 c			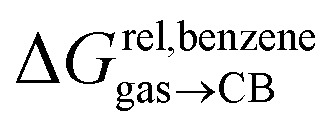 d	
	Probe P3		Probe P1	Probe P3		Probe P1	Probe P3		Probe P1	Probe P3
Cyclopentane	22.1	(±0.6)	14	9.9	(±0.3)	−1.56	−1.36	(±0.02)	0.46	0.66
Cyclopentene	4.7	(±0.2)	5.6	2.1	(±0.1)	−1.02	−0.44	(±0.03)	0.28	0.86
Cyclohexane	244	(±4)	160	110	(±2)	−3.01	−2.78	(±0.01)	−1.04	−0.82
Cyclohexene	38.0	(±0.6)	14	17.0	(±0.3)	−1.56	−1.68	(±0.01)	−0.46	−0.58
1,3-Cyclohexadiene	6.3	(±0.5)	2.2	2.8	(±0.2)	−0.47	−0.62	(±0.05)	−0.05	−0.20
1,4-Cyclohexadiene	13	(±1)	4.2	5.8	(±0.4)	−0.85	−1.04	(±0.04)	−0.57	−0.76
Benzene	2.2	(±0.1)	1.0	1.00	(±0.05)	0.00	0.00	(±0.03)	0.00	0.00
Cycloheptene	1000		140	448		−2.93	−3.62		−2.02	−2.71
Cyclooctatetraene	747	(±71)	5.6	334	(±32)	−1.02	−3.44	(±0.06)	−1.97	−4.40

a Binding affinity relative to cycloheptene (set to 1000).

b Relative binding affinity normalized to the affinity of benzene.

c Free energy of hydrocarbon transfer from aqueous solution to the cavity of the CB[8]/auxiliary probe complexes; in kcal mol^−1^ and normalized to benzene.

d Free energy of hydrocarbon transfer from the gas phase (molar reference state) to the cavity of the CB[8]/auxiliary probe complexes in solution; in kcal mol^−1^ and normalized to benzene.

## Results

We prepared probe P3 as a minimalist mimic of probe P1, from 4-methylbenzylbromide and 4-(trifluoromethyl)pyridine in 60% yield. Addition of 0.50 equiv. CB[8] affords homoternary complex CB[8]·P3_2_ quantitatively (see Fig. 1, spectra a and b). Large upfield shifts of 0.76, 1.39 and 1.32 ppm for protons H^b^, H^c^ and H^d^, respectively, were observed, along with proton H^a^ (0.34 ppm), confirming the encapsulation of the tolyl unit and methylene moiety within the CB[8] cavity. Conversely, the ^19^F NMR signal shifted downfield by 0.29 ppm, as expected, due to the position of the trifluoromethyl group in the deshielding environment of the CB[8] carbonyl rim upon complexation. Addition of a small excess of CB[8] (1.0 equiv.) and an excess amount of hydrocarbon H followed by sonication afforded, again quantitatively, heteroternary complexes CB[8]·P3·H (see Fig. 1, spectra c for H = cyclohexane, and ESI† section for other hydrocarbons). This stands in contrast to assemblies CB[8]·P1·H which remained at equilibrium with the homoternary precursor CB[8]·P1_2_.^11^ Significant downfield ^19^F NMR shifts were observed with all hydrocarbons (0.46–0.64 ppm; 0.58 ppm for cyclohexane, see Fig. 1). Optimization of complexes CB[8]·P3·H with the recently developed GFN2-xTB semi-empirical method^14–16^ in conjunction with the ALPB solvation model^17^ suggests that the pyridinium unit acts as a lid for one of the CB[8] portals (see Fig. 1); reoptimization after a 180° rotation of the lid along the C_aryl_–CH_2_ bond away from the portal consistently destabilizes the assembly (by 3.5 kcal mol^−1^ when cyclohexane is encapsulated, for example).

Binding affinities of hydrocarbons to assembly CB[8]·P1, relative to a reference hydrocarbon, were calculated using the ratio of homo- and heteroternary complexes CB[8]·P1_2_ and CB[8]·P1·H.^11^ As hydrocarbons bind assembly CB[8]·P3 quantitatively, we used competitive binding experiments to determine relative binding affinities 
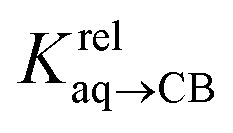
, by varying the ratio of two hydrocarbons H and H′ added in excess to a solution of homoternary complex CB[8]·P3_2_, and by determining the ratio of heteroternary assemblies CB[8]·P3·H and CB[8]·P3·H′ in solution (see equilibrium (1) and eqn (2)).

1 CB[8]·P3·H + H′ ⇄ CB[8]·P3·H′ + H

2
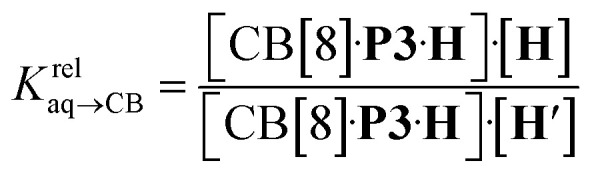


**Fig. 2:**
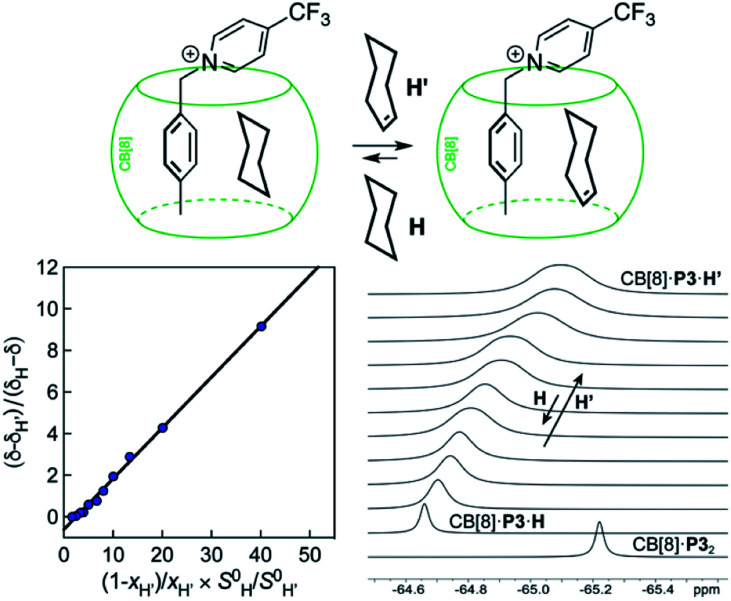
Competition between cyclohexane and cycloheptene for assembly CB[8]·P3 in deuterium oxide. Plot of (*δ*–*δ*_H′_)/(*δ*_H_–*δ*) as a function of 
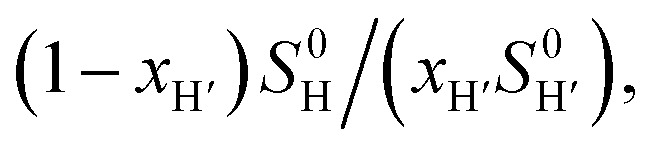
 where *δ*_H_ is the ^19^F chemical shift of complex CB[8]·P3·cyclohexane, *δ*_H′_ the ^19^F chemical shift of complex CB[8]·P3·cycloheptene, and *δ* chemical shifts of mixtures thereof; *S*^0^ and *x* are the hydrocarbon solubilities and molar fractions of each hydrocarbon in the mixture. ^19^F NMR shifts *δ* measured upon addition of cycloheptene to a solution of ternary complex CB[8]·P3·cyclohexane.

The concentrations of free hydrocarbons H and H′ in solution are their solubility in water. In an ideal mixture of two or more solutes, the solubility of a solute *i* is obtained from eqn (3) where *x*_*i*_ is the molar fraction of solute *i* in the mixture and *S*^0^_*i*_ the solubility of pure solute *i*. As both hydrocarbons exchange fast on the ^19^F NMR time scale (see Fig. 2), the ratio of both heteroternary complexes can be obtained from the observed chemical shift *δ* during the competition experiment, and the chemical shifts *δ*_H_ and *δ*_H′_ corresponding to pure heteroternary complexes CB[8]·P3·H and CB[8]·P3·H′, respectively. Therefore eqn (3) can be rewritten as eqn (4).

3 *S*_*i*_ = *x*_*i*_*S*^0^_*i*_

4
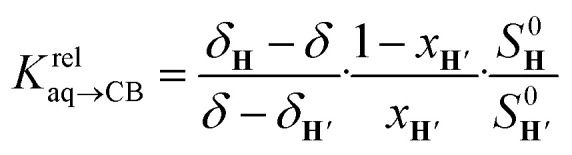


The relative binding affinity 
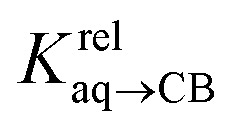
 is then obtained from the slope of the best straight line in a plot of (*δ*–*δ*_H′_)/(*δ*_H_–*δ*) as a function of 
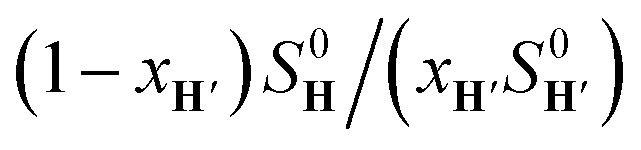
 (see Fig. 2 when hydrocarbons H and H′ are cyclohexane and cycloheptene, respectively). We opted not to force the straight line through origin to account for the non-ideality of the hydrocarbon mixture; the impact on the relative affinities is insignificant (see Table S1†). The free energy term 
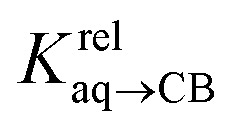
 that corresponds to the transfer of the hydrocarbon from solution to the tailor-made cavity of assembly CB[8]·P3, relative to a reference hydrocarbon, is obtained from eqn (5) (see Table 1).5
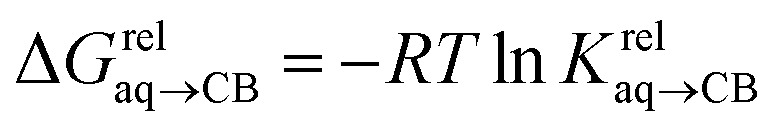


**Fig. 3:**
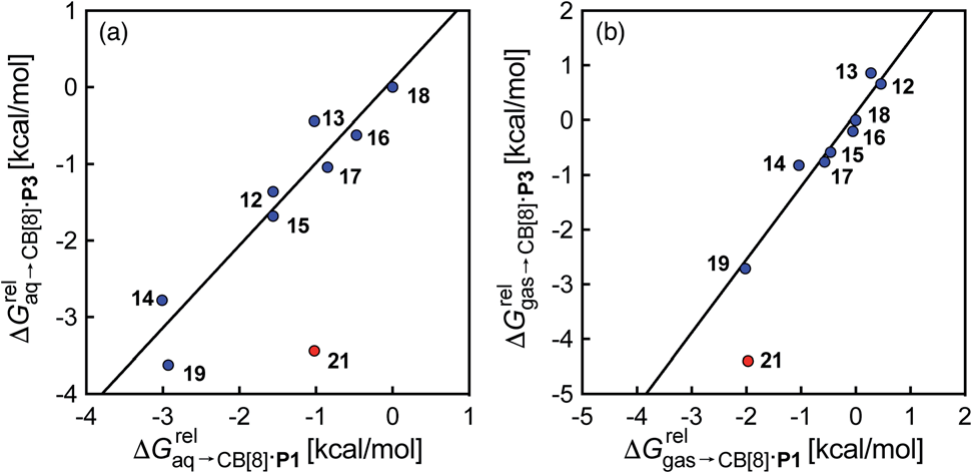
Comparison of the relative free energies of hydrocarbon binding to assembly CB[8]·P3 and CB[8]·P1, using hydrocarbons (a) in aqueous solution, and (b) in the gas phase. See Table 2 for hydrocarbon numbering; outlier highlighted in red.

Pairs of hydrocarbons were chosen to maximize ^19^F NMR chemical shift differences between assemblies CB[8]·P3·H and CB[8]·P3·H′. Cycloheptene was used as reference in most cases, except for 1,4-cyclohexadiene and cyclooctatetraene that were combined with cyclohexene and cyclohexane, respectively (*i.e.* the latter two hydrocarbons are used as relays). Table 1 presents binding affinities normalized to cycloheptene (
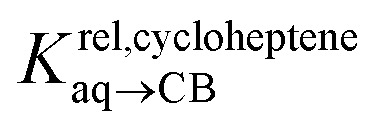
) and benzene (
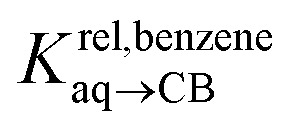
), respectively. Despite the obvious structural difference between probes P1 and P3 and a different analytical treatment, relative binding affinities of hydrocarbons to assemblies CB[8]·P1 and CB[8]·P3 are remarkably similar (see Table 1 and Fig. 3a). Like assembly CB[8]·P1, binary complex CB[8]·P3 binds preferentially to saturated hydrocarbons. For example, cyclohexane, cyclohexene, 1,3- and 1,4-cyclohexadiene bind 110, 17, 2.8 and 5.8 times better than benzene (see Table 1); similarly, the affinity of cyclopentane is 5 times higher than cyclopentene. The only pronounced difference between both systems appears with cyclooctatetraene, which binds assembly CB[8]·P3 330 times better than benzene, and assembly CB[8]·P1 only 5.6 times better than benzene.

## Discussion

The transfer of the hydrocarbon from aqueous solution to the cavity of assembly CB[8]·P3 can be separated into a desolvation (or dehydration) term and the interaction between the hydrocarbon and the cavity. We highlighted in our 2017 study^11^ that the free energy of solvation of a solute *i* in a given solvent is readily obtained from its solubility *S*_*i*_ in that solvent and its vapor pressure *P*_vap,*i*_ using eqn (6).6
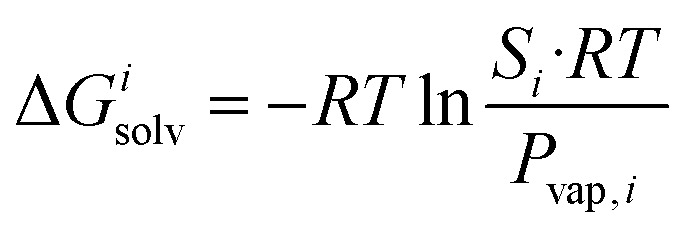


This relationship is equivalent to the one later proposed by Gilson, Grimme and Nau,^10^ and Nau and Scherman^12^ (see eqn (7)), where *P*^0^ is 101.325 kPa (*i.e.* 1 atm). The −1.90 kcal mol^−1^ correction term corresponds to the change of reference state in the gas phase from 1 atm to 1 M (*i.e.* 1 mol of gas per liter of gas, or 24.5 atm; see demonstration in the ESI† section).7
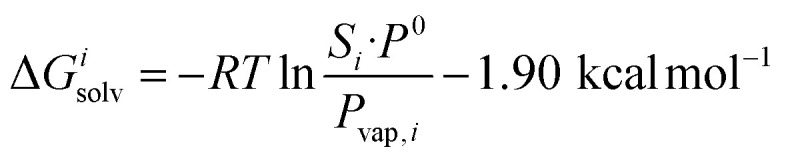


Since solvation energies in water 
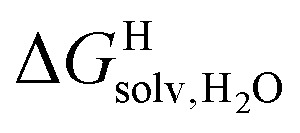
 are only known for a fraction of hydrocarbons used in this study, we obtained them *in silico* using density functional theory, the highly accurate COSMO-RS model and the Cosmotherm software (see ESI† section for details). Considering the experimental challenges associated with solubility measurements, the linear correlation observed between calculated and tabulated solvation energies is excellent (*R*^2^ = 0.95, see Fig. S13 and ESI† section for details). The free energy of binding 
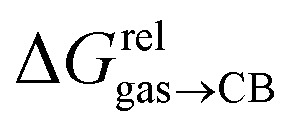
 between the desolvated hydrocarbon and the cavity of assembly CB[8]·P3 (*i.e.* the free energy of transfer from the gas phase to the cavity) was then obtained from eqn (8) (see Table 1), where 
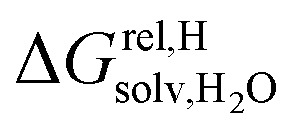
 is the hydrocarbon solvation energy in water relative to a reference hydrocarbon (benzene in Table 1).8
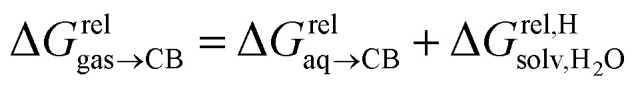


Again, with the exception of cyclooctatetraene, assemblies CB[8]·P1 and CB[8]·P3 display strikingly similar trends in binding affinities towards hydrocarbons (see Table 1 and Fig. 3b). A plot of the relative free energies of binding 
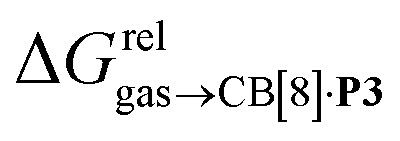
 as a function of 
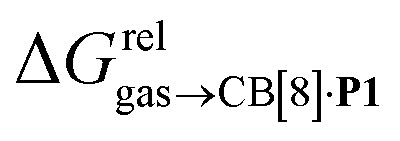
 affords a straight line with a slope of 1.3 (±0.1), thereby indicating a slight enhancement in hydrocarbon selectivity with CB[8]·P3 compared to CB[8]·P1.

**Fig. 4:**
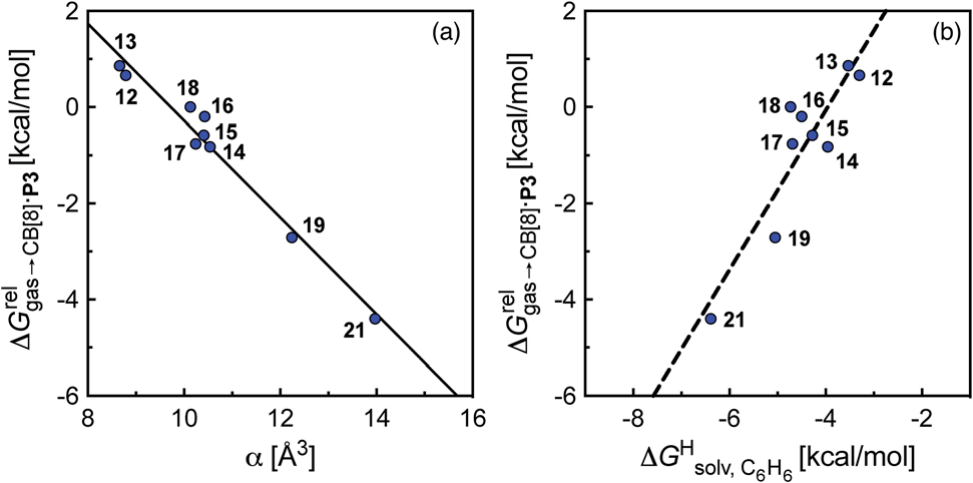
Relative free energies of transfer of hydrocarbons from the gas phase to the cavity of assembly CB[8]·P3 (
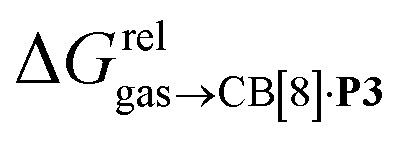
) as a function of (a) the hydrocarbon polarizability *α* [Å^3^], and (b) the free energy of solvation of the hydrocarbons in benzene 
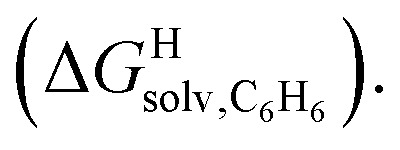
 See Table 2 for hydrocarbon numbering.

To justify these trends, we attempted to identify correlations between the free energy of binding 
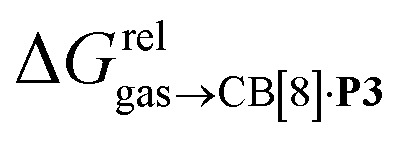
 and various physicochemical descriptors of each hydrocarbon, including (1) their volume, calculated with the PM6 semi-empirical model and delimited by a 0.002 electron per Bohr^3^ isodensity surface (see Table 2), (2) their solvent accessible surface area (obtained with the same method), and (3) their static polarizability *α* (see Table 2), calculated by DFT at the very accurate^18^ pbe0/aug-cc-pVTZ level^19–21^ after successful comparison with experimental values (see Fig. S14;† a plot of experimental *vs.* calculated polarizabilities returns a coefficient of determination *R*^2^ of 0.997). While no clear trend was obtained with the first two descriptors (see Fig. S15†), a remarkably linear correlation was obtained between the 
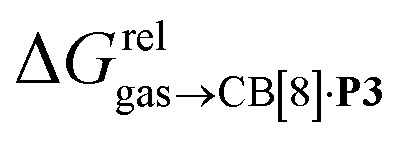
 terms and the polarizabilities *α* of the hydrocarbons (*R*^2^ = 0.97, see Fig. 4a). A similar correlation, albeit of poorer quality (*R*^2^ = 0.85), was observed by Gilson, Grimme and Nau for 26 hydrocarbons and perfluoroalkanes binding to CB[7].^10^ We tested the relationship again using only the 15 rigid hydrocarbons listed in Table 1 (*i.e.* butane, pentane, hexane and others were removed as additional entropic penalties for limiting rotational freedom upon CB[7] binding might bias results); the trend persisted, and the quality of the linear regression improved (*R*^2^ = 0.92; see Fig. S16†). The linear correlation between relative free energies of binding 
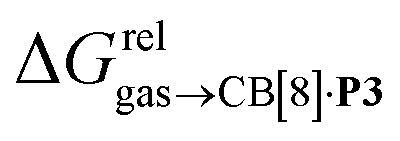
 and hydrocarbon polarizabilities observed with assembly CB[8]·P3 strongly suggests that the selectivity is driven, perhaps exclusively, by dispersive interactions between the hydrocarbons and the tailor-made cavity, regardless of the geometry, or degree of unsaturation of the guests, or weak electrostatic host–guest interactions.

**Table 2:** Physicochemical and thermodynamic properties of hydrocarbons, as well as the CB[6]-, CB[7]-, CB[8]·P2- and CB[8]·P3-hydrocarbon complexes assessed in this work

	Hydrocarbon	*V* a	*σ*′^b^	*α* c	Δ*G*^H^_solv_ d			Δ*G*^H^_cav_ e		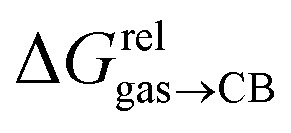 f			
					H_2_O	C_6_H_6_	C_6_F_14_	C_6_H_6_	C_6_F_14_	CB[6]	CB[7]	CB[8]·P2	CB[8]·P3
1	Methane	33.7	3.70	2.50	1.39	−0.18	0.39	4.89	1.51		−3.35	1.94	
2	Ethane	53.6	4.32	4.27	1.61	−0.97	−0.18	6.58	1.93	−4.37	−3.21	2.02	
3	Ethene	45.1	4.07	4.10	0.85	−1.11	−0.21	5.88	1.76		−3.30		
4	Acetylene	36.6	3.80	3.44	−0.34	−1.27	−0.13	5.14	1.57		−3.21		
5	Propane	73.3	4.79	6.08	1.77	−1.64	−0.67	8.11	2.31	−5.40	−3.38	0.21	
6	Propene	64.8	4.60	5.99	0.85	−1.89	−0.75	7.46	2.15		−3.34		
7	*Cis*-butene	84.4	5.02	7.85	0.79	−2.73	−1.36	8.94	2.51	−6.27	−5.41	−2.80	
8	*Trans*-butene	84.5	5.02	7.93	1.06	−2.63	−1.32	8.95	2.51		−4.60	−0.82	
9	Isobutane	92.7	5.18	7.88	1.82	−2.24	−1.10	9.55	2.66	−6.27	−5.58	−0.21	
10	Isobutene	84.3	5.02	7.84	0.83	−2.54	−1.20	8.93	2.51	−5.89	−5.49	−1.64	
11	Neopentane	111.8	5.51	9.67	1.81	−2.76	−1.48	10.91	2.98		−6.38	−3.39	
12	Cyclopentane	99.7	5.31	8.79	1.07	−3.30	−2.05	10.06	2.78	−7.26	−6.15	−1.42	0.66
13	Cyclopentene	91.5	5.16	8.66	0.36	−3.53	−2.13	9.46	2.64	−6.66		−2.93	0.86
14	Cyclohexane	118.5	5.62	10.54	1.02	−3.96	−2.57	11.38	3.09		−7.41	−1.46	−0.82
15	Cyclohexene	110.2	5.49	10.41	0.16	−4.28	−2.68	10.80	2.95				−0.58
16	1,3-Cyclohexadiene	101.9	5.34	10.43	−0.52	−4.50	−2.73	10.21	2.81			−4.24	−0.20
17	1,4-Cyclohexadiene	102.0	5.35	10.24	−0.67	−4.69	−2.89	10.22	2.82				−0.76
18	Benzene	93.3	5.19	10.13	−0.94	−4.73	−2.89	9.59	2.67		−6.71	−3.99	0.00
19	Cycloheptene	129.1	5.78	12.24	−0.04	−5.05	−3.26	12.11	3.26				−2.71
20	Norbornene	117.8	5.61	11.30	−0.04	−4.66	−2.96	11.33	3.08		−7.89		
21	Cyclooctatetraene	123.9	5.70	13.97	−1.90	−6.39	−4.00	11.75	3.18				−4.40

a Hydrocarbon volume calculated with the PM6 semi-empirical model and delimited by a 0.002 electron per Bohr^3^ isodensity surface; in Å^3^.

b Effective hard sphere diameter obtained from eqn (12); in Å.

c Static polarizability calculated at the pbe0/aug-cc-pVTZ level; in Å^3^.

d Free energies of solvation in water, benzene and perfluorohexane, calculated with the COSMO-RS solvation model and the Cosmotherm software; in kcal mol^−1^.

e Cavitation energies in benzene and perfluorohexane, obtained from eqn (10)–(12); in kcal mol^−1^.

f Free energies of transfer from the gas phase (molar reference state) to the cavities of CB[6], CB[7], CB[8]·P2 and CB[8]·P3 in aqueous solution; in kcal mol^−1^.

The correlation between energy terms and static polarizabilities (measured either in units of volume (Å^3^) or in C^2^ m^2^ J^−1^) did not really satisfy us, however. For example, what is the physical meaning of the slope of the regression? A correlation between two energy terms would certainly be far more informative. We thus questioned whether the solvation energy afforded to the hydrocarbons by the cavity in assembly CB[8]·P3 (*i.e.* on one side the CB[8] inner wall, and on the other a tolyl unit) could be mimicked and reproduced by the solvation offered by a simple non-polar solvent such as benzene. Perhaps unsurprisingly, a plot of 
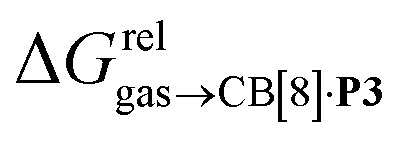
 as a function of the free solvation energy of the hydrocarbons in benzene 
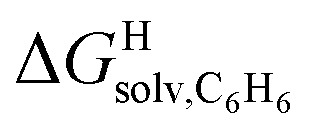
 (calculated again with the COSMO-RS solvation model and the Cosmotherm software) only afforded a mediocre linear regression with a slope of 1.7 (±0.3) and a coefficient of determination *R*^2^ = 0.82 (see Fig. 4b).

Based on the work by Ben Amotz^22–25^ and Schmid,^26,27^ we then sought to separate the hypothetical solvation of the hydrocarbons in a non-polar solvent into two terms (see eqn (9)): (1) the free (repulsive) cavitation energy Δ*G*^H^_cav_ required to form a cavity inside the solvent to accommodate the solute, and (2) the free (attractive) and mainly dispersive energy Δ*G*^H^_disp_ released upon introduction of the solute into the cavity.

9 Δ*G*^H^_solv_ = Δ*G*^H^_cav_ + Δ*G*^H^_disp_

As a linear correlation is observed between 
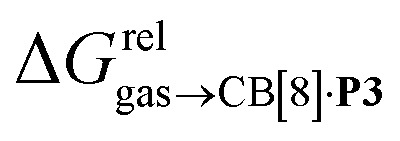
 and polarizabilities, and as dispersive interactions are favorable between polarizable units, we sought to test whether 
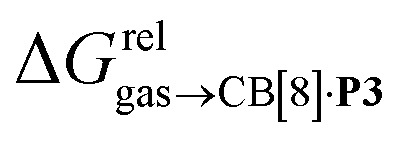
 might correlate with the Δ*G*^H^_disp_ term, *i.e.* with Δ*G*^H^_solv_ − Δ*G*^H^_cav_. While 
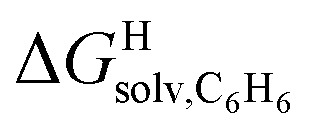
 is readily available from COSMO-RS calculations, the cavitation energy 
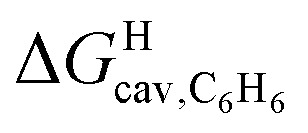
 must be approximated. Within the hard fluid model, the cavitation energy required to accommodate a hard sphere solute in a hard sphere solvent can be obtained by a variation of the Boublik–Mansoori–Carnahan–Starling–Leland equation of state^28,29^ proposed by Matyushov and Ladanyi^23,24,28–30^ (see eqn (10)).10
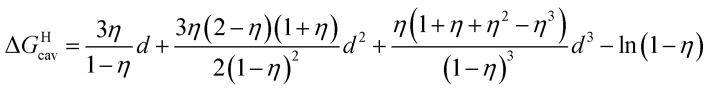


In eqn (10), *d* is the solute–solvent diameter ratio *σ*_solute_/*σ*_solvent_, and *η* is the solvent packing fraction obtained from eqn (11) (*N*_A_ being the Avogadro constant, *σ*_solvent_ the diameter of the solvent as a hard sphere expressed in Å^3^, *ρ* its density in g cm^−3^ and *M* its molar mass in g mol^−1^).11
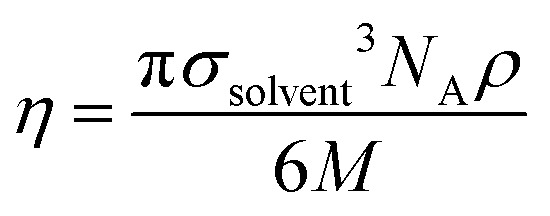


The diameter of the hard spheres was obtained from eqn (12). The empirical coefficient *c* (0.922) originates from the small difference between the diameter obtained from our volumes delimited by isodensity surfaces, and *σ* diameters tabulated by Ben Amotz for a subset of solvents^22^ (see Fig. S17.† This calibration is important, as the Δ*G*^H^_cav_ term is highly dependent on the size of the solvent hard sphere. The calibration is highlighted with the parameter *σ*′, to contrast with tabulated *σ* diameters.^22^12
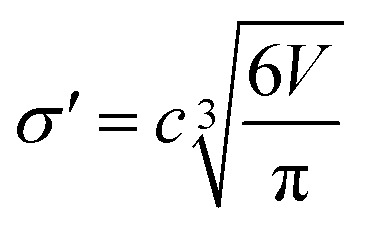


Eqn (12) returns cavitation energies in benzene 
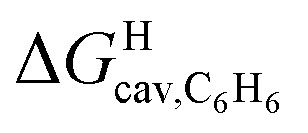
 ranging from 9.5 kcal mol^−1^ for cyclopentene to 12.1 kcal mol^−1^ for cycloheptene (see Table 2).

**Fig. 5:**
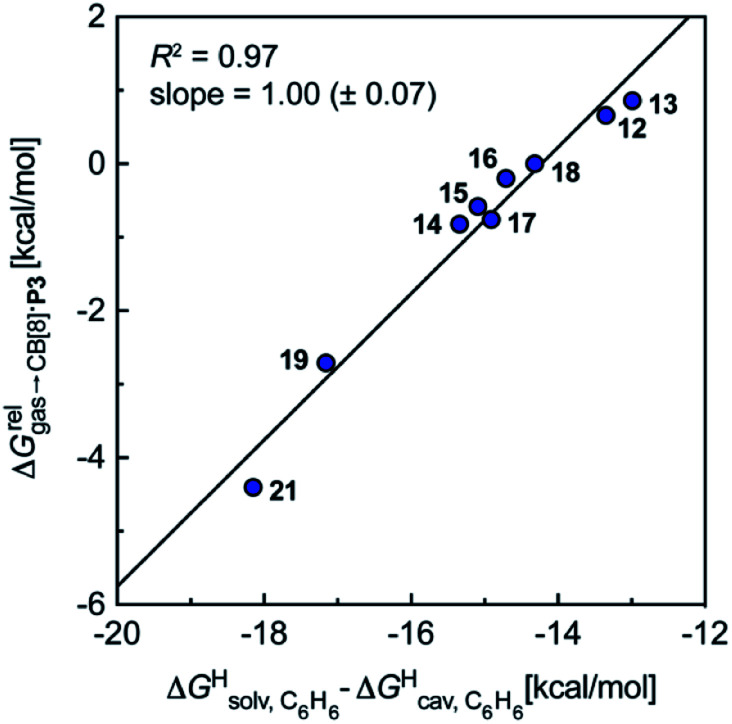
Relative free energies of transfer of hydrocarbons from the gas phase to the cavity of assembly CB[8]·P3
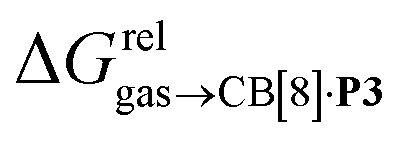
 as a function of the energy released upon introduction of the hydrocarbon into a pre-formed cavity in benzene 
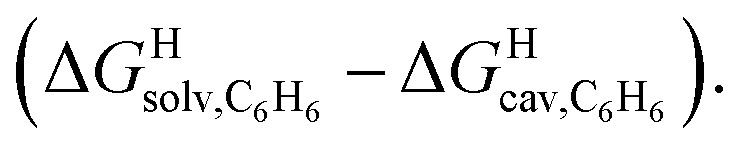
 See Table 2 for hydrocarbon numbering.

Remarkably, the free energies of transfer of the hydrocarbons from the gas phase to the cavity of assembly CB[8]·P3
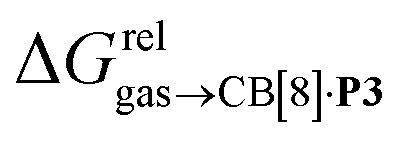
 correlate linearly with 
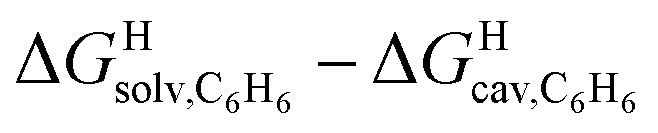
 (*i.e.*
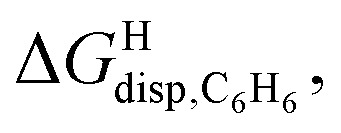
 see eqn (9)) with a slope of 1.00 (±0.07) and a coefficient of determination *R*^2^ = 0.97 (see Fig. 5)! This result leads to the following empirical conclusion: assembly CB[8]·P3 behaves as a non-polar, yet polarizable solvent (benzene) that does not suffer any energetic penalty for the formation of the cavity that accommodates the hydrocarbon guests; in other terms, the cavity is pre-formed, as long as it allows the guest to fit in.

**Fig. 6:**
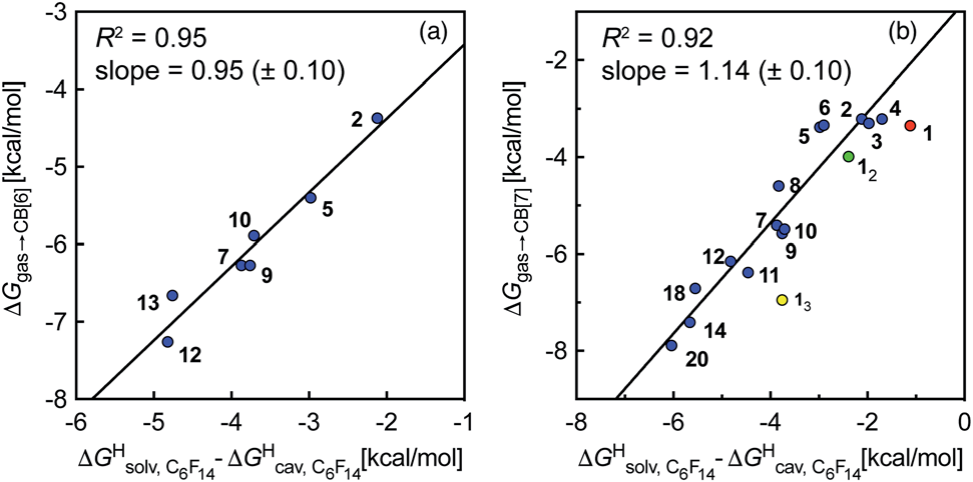
Free energies of transfer of hydrocarbons from the gas phase to the cavity of (a) CB[6] (Δ*G*_gas→CB[6]_) and (b) CB[7] (Δ*G*_gas→CB[7]_) as a function of the energy released upon introduction of the hydrocarbon into a pre-formed cavity in perfluorohexane 
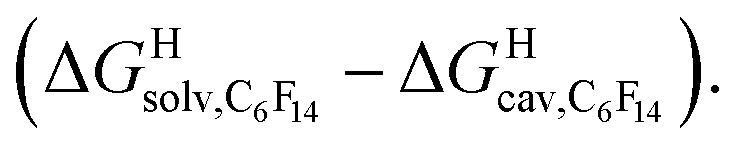
 See Table 2 for hydrocarbon numbering.

To test the scope of this model, we attempted to correlate the free energies of transfer of hydrocarbons from the gas phase to the cavity of CB[6]^7,12^ Δ*G*_gas→CB[6]_ with Δ*G*^H^_solv_ − Δ*G*^H^_cav_. When CB[6] is mimicked by benzene, a linear correlation is obtained (*R*^2^ = 0.97), but with a slope of only 0.46 (±0.04) (see Fig. S18†). However, as shown by Nau and coworkers the cavity of CB[*n*]s is highly non-polar and non-polarizable – in fact, the polarizability of CB[7] is even weaker than perfluorohexane.^8,31–36^ We thus calculated Δ*G*^H^_solv_ − Δ*G*^H^_cav_ for perfluorohexane instead of benzene (see Table 2), and checked again for a correlation with Δ*G*_gas→CB[6]_. This time, while the coefficient of determination remains very high (*R*^2^ = 0.95), the slope of the linear regression reaches 0.95 (±0.10)! One can thus conclude that our empirical model can even predict the very low, perfluorohexane-like polarizability of the CB[6] cavity (see Fig. 6a).

We then attempted a similar correlation using the free energies of transfer of hydrocarbons from the gas phase to the cavity of CB[7]^8,10,12^ and Δ*G*^H^_solv_ − Δ*G*^H^_cav_ for perfluorohexane. A slope of 1.14 (±0.10) was obtained with a good coefficient of determination (*R*^2^ = 0.92; see Fig. 6b). The near unity slope suggests that our model remains valid for CB[7]. One notable outlier is methane (1, highlighted in red in Fig. 6b), whose affinity is stronger than predicted. As CB[7] should be suspected to encapsulate more than one methane molecule, we optimized the structure of a putative methane dimer using DFT at the TPSS-D3(BJ)/def2-TZVP level, and treated it as a standalone guest for CB[7] (see ESI† section for details). An excellent alignment with the regression line is then obtained (see green data point labeled 1_2_ in Fig. 6b), after correction for the free energy of the endergonic dimerization reaction (+1.76 kcal mol^−1^). A similar calculation with a putative cyclic methane trimer (free energy of the trimerization +5.57 kcal mol^−1^) shows a large deviation from the model (see yellow data point labeled 1_3_ in Fig. 6b). An even larger deviation is observed with a putative methane/water heterodimer (see Fig. S20†), thereby strongly suggesting that CB[7] encapsulates two methane molecules on average; we do note that this hypothesis will have to be verified experimentally.

The model was then tested with assembly CB[8]·P1. Relative free energies of hydrocarbon binding 
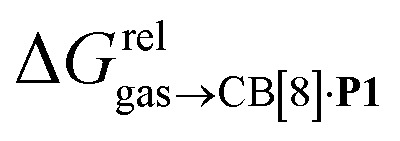
 as a function of the energy released upon introduction of the hydrocarbon into pre-formed cavities in benzene 
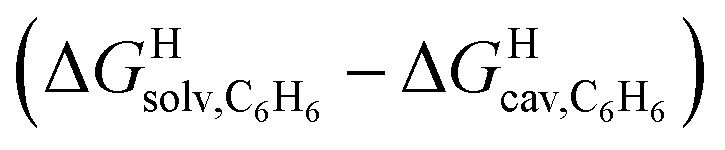
 and perfluorohexane 
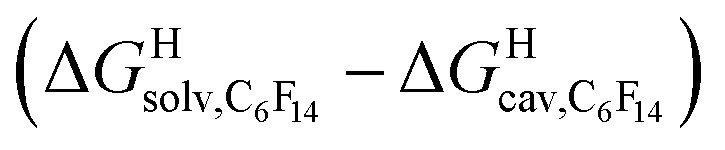
 afforded straight lines with slopes of 0.60 (±0.08) and 1.3 (±0.2), respectively (see Fig. S19†). One concludes that the cavity available for hydrocarbon binding in assembly CB[8]·P1 has a polarity and polarizability between those of benzene and perfluorohexane. The lower coefficients of determination (0.91 and 0.83, respectively) also suggest that these two solvents are perhaps not ideal mimics of the cavity.

**Fig. 7:**
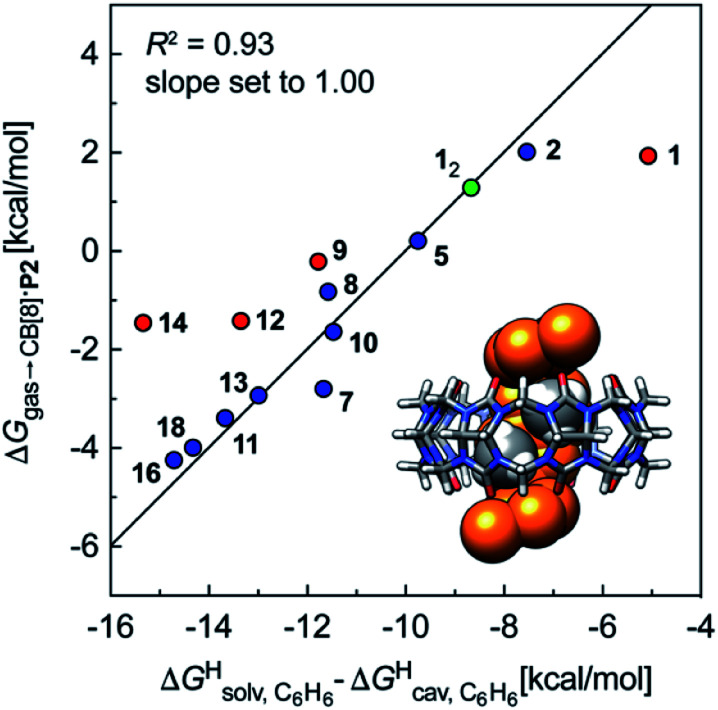
Free energies of transfer of hydrocarbons from the gas phase to the cavity of assembly CB[8]·P2 Δ*G*_gas→CB[8]·P2_ as a function of the energy released upon introduction of the hydrocarbon into a pre-formed cavity in benzene 
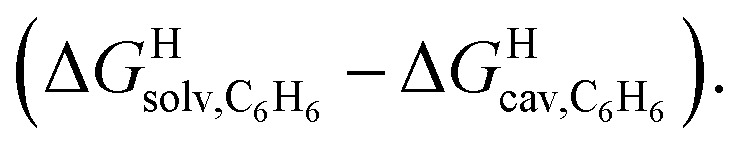
 See Table 2 for hydrocarbon numbering. Outliers in red.

We also tested the model with Nau's and Scherman's assembly CB[8]·P2 and Δ*G*^H^_solv_ − Δ*G*^H^_cav_ terms for benzene, as auxiliary guest P2 contains an aromatic core (see Fig. 7). Although the correlation using the complete set of hydrocarbons is not satisfactory, it highlights an important element of our model: it is valid as long as the guest can fit into the cavity; if the guest is too large, repulsive forces become overwhelming, and affinities drop precipitously. Forcing the slope of the regression to 1.0 highlights four outliers (in red in Fig. 7): (a) cyclohexane (14) and cyclopentane (12) whose affinities are weaker than predicted; we suspect that these two guests are simply too large to fit in the cavity; (b) methane (1), which again seems to bind too strongly; but again, using a methane dimer returns a data point sharply in line with the model (see green data point labeled 1_2_ and optimized structure of the complex in Fig. 7); (c) at this time, we cannot justify the weaker than expected affinity of isobutane (9). After exclusion of cyclohexane and cyclopentane, the linear correlation is very satisfactory (*R*^2^ = 0.93).

**Fig. 8:**
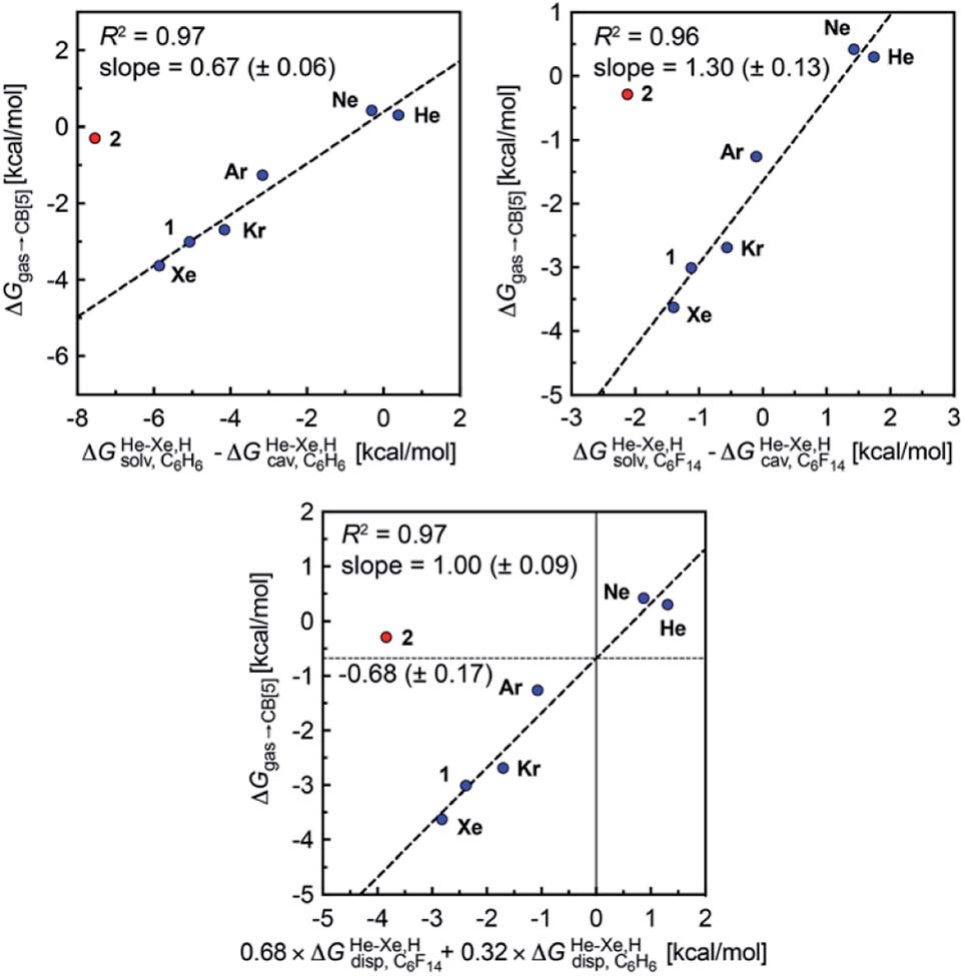
Free energies of transfer of noble gases (He–Xe), methane (1) and ethane (2) from the gas phase to the cavity of assembly CB[5] Δ*G*_gas→CB[5]_ as a function of the energy released upon introduction of the guests into a pre-formed cavity in benzene 
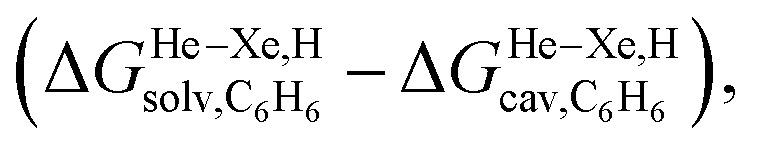
 perfluorohexane 
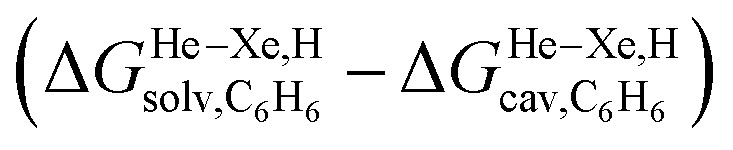
 and a 68 : 32 mixture of perfluorohexane and benzene. See Table 2 for hydrocarbon numbering. The ethane (2) outlier is highlighted in red.

Finally, we questioned whether the model might be applicable to noble gases binding to CB[5]. Nau and coworkers showed that He, Ne, Ar, Kr and Xe bind to CB[5] with affinities of 87, 72, 360, 2400 and 8700 M^−1^, respectively.^37^ The affinities of methane and ethane are 210 and 24 M^−1^. The authors show very convincingly that (1) the main driving force of the encapsulation is the release of cavitation energy 
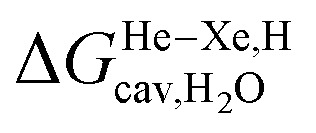
 when the guests transfer from bulk water to the CB[5] cavity (*i.e.* when they leave a “hole” in water that collapses into new water–water interactions); and (2) dispersive interactions are stronger between guests and water than between guests and CB[5], *i.e.* the encapsulation would be unfavourable were it not for the release of cavitation energy. All cavitation and dispersive terms increase as the volume of the guests increases, but to different extents. We show first that free energies of guest transfers from the gas phase to the CB[5] cavity in aqueous solution Δ*G*_gas→CB[5]_ calculated using eqn (8) and the COSMO-RS solvation model for the solvation term 
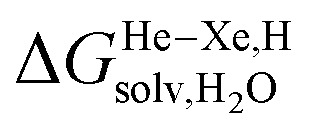
 are comparable to those obtained by Nau and coworkers using the CSM-D solvation model^38,39^ (see Table 3). Our empirical model then returns linear correlations between free energies of transfer Δ*G*_gas→CB[5]_ and Δ*G*^He–Xe,H^_solv_ − Δ*G*^He–Xe,H^_cav_ terms for both benzene and perfluorohexane (*R*^2^ = 0.97 and 0.96, and slopes of 0.60 (±0.06) and 1.30 (±0.13), respectively, see Fig. 8), after removal of the ethane (2) outlier that is too large to fit into CB[5]. Like CB[8]·P1, the polarity and polarizability of the CB[5] cavity lies between these two solvents; a straight line with a unity slope is obtained with a 68 : 32 mixture of perfluorohexane and benzene (see Fig. 8), assuming additivity of the dispersive terms (see eqn (13), where *x*_C_6_H_6__ and *x*_C_6_F_14__ are the molar fractions of both solvents).13
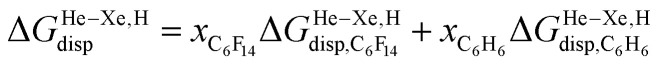


**Table 3:** Thermodynamic properties of noble gases (He–Xe), methane (1) and ethane (2) and their CB[5] complexes

	Δ*G*^He–Xe,H^_solv_ a			Δ*G*^He–Xe,H^_cav_ b		Δ*G*^He–Xe,H^_disp_ c	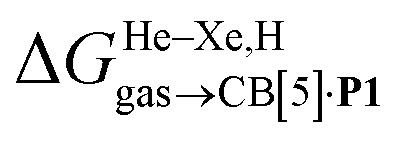 d
	H_2_O	C_6_H_6_	C_6_F_14_	C_6_H_6_	C_6_F_14_	C_6_F_14_/C_6_H_6_ 68 : 32	
He	2.90	2.54	2.51	2.15	0.77	+1.31 (+1.8)	+0.30 (+0.1)
Ne	2.92	2.33	2.33	2.64	0.91	+0.87 (+0.5)	+0.42 (+0.1)
Ar	2.24	0.93	1.20	4.09	1.30	−1.07 (−1.4)	−1.26 (−1.5)
Kr	1.91	0.53	0.89	4.67	1.45	−1.70 (−3.2)	−2.69 (−3.0)
Xe	1.77	−0.19	0.31	5.66	1.70	−2.82 (−4.9)	−3.63 (−4.1)
1	1.39	−0.18	0.39	4.89	1.51	−2.38 (−2.3)	−3.01 (−2.4)
2	1.61	−0.97	−0.18	6.58	1.93		−0.29 (−0.1)

a Free energies of solvation in water, benzene and perfluorohexane, calculated with the COSMO-RS solvation model and the Cosmotherm software; in kcal mol^−1^.

b Cavitation energies in benzene and perfluorohexane, obtained from eqn (10)–(12); in kcal mol^−1^.

c Dispersion energy term of noble gases, methane and ethane interacting with a 68 : 32 mixture of perfluorohexane and benzene, obtained from eqn (9) and (13), in kcal mol; in parenthesis: from ref. 37 calculated for the CB[5] complexes at the TPSS-D3/def2-TZVP level.

d Free energies of transfer from the gas phase (molar reference state) to the cavity of CB[5] in aqueous solution, obtained from association constants in ref. 37 and COSMO-RS solvation energies calculated herein (and, in parenthesis, calculated with the CSM-D solvation model^38,39^ reported in ref. 37).

The dispersive interactions Δ*G*^He–Xe,H^_disp_ (*i.e.* Δ*G*^He–Xe,H^_solv_ − Δ*G*^He–Xe,H^_cav_) between the guests and this mixture of solvents are similar to those calculated with CB[5] by Nau and coworkers at the TPSS-D3/def2-TZVP level (−2.8 to +1.3 kcal mol^−1^*vs.* −4.9 to +1.8 kcal mol^−1^ from Xe to He, see Table 3). The *y*-intercept (*i.e.* when free dispersive interactions Δ*G*^He–Xe,H^_disp_ are absent, see Fig. 8) corresponds to the cavitation energy 
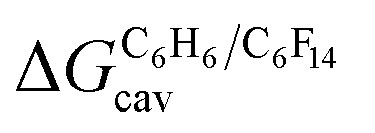
 required to create a void inside the solvents mimicking the cavity of CB[5] to accommodate the guests. As hypothesized by Nau and coworkers, the CB[5] cavity is very weakly hydrated, and Δ*G*^CB[5]^_cav_ should be near zero; our model correctly returns 
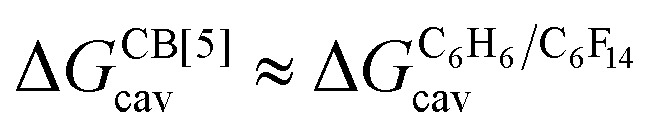
 equal to only −0.68 (±0.17) kcal mol^−1^ (see Fig. 8). Therefore, while our model in its present form does not predict absolute binding affinities for CB[6], CB[7] and CB[8]/auxiliary probe systems, it does for CB[5] as Δ*G*^CB[5]^_cav_ ≈ 0! It is widely known^1–5,33,34,40,41^ that the main driving force of guest encapsulation into CB[6]–CB[8] is the ejection of water from the cavity back to bulk water. This energy term is embedded into the *y*-intercept values of our model's regression lines, that are unique to each system studied so far.

## Conclusions

The serendipity of an excellent correlation between hydrocarbon polarizabilities and their free energies of transfer from the gas phase to assembly CB[8]·P3 allowed us to create a new empirical model for hydrocarbons, and even noble gases, binding to CB[*n*]-based cavities that does not even implicate CB[*n*]s. We propose those cavities behave as low (and tunable) polarity and polarizability solvents with a pre-formed cavity that accommodates the guests without any cavitation penalty, as long as the guests can fit inside the cavity. The model appears valid for CB[5], CB[6], CB[7] and at least the pair of simple CB[8]/auxiliary probe assemblies CB[8]·P2 and CB[8]·P3, and provides a computationally expedient solution to predict the selectivity of hydrocarbons to CB[*n*]-based cavities. The scope and limitations of the model will be assessed with other guests (neutral and positively charged) and other macrocycles in subsequent studies.

## Data availability

All analytical data is provided in the narrative and in the ESI.† All details about computational methods and associated coordinates are available in the ESI.†

## Author contributions

EM conceived the project, designed the model, performed all calculations, and mentored MN, HB and RR who carried out all the experimental work. EM, HB and MN wrote the manuscript.

## Conflicts of interest

There are no conflicts to declare.

## Supplementary Material

SC-013-D1SC06728A-s001
