# Supplementary material for: Cucurbiturils mimicked by low polarizability solvents with pre-formed cavities: an empirical model to predict hydrocarbon selectivity
Source: Chem Sci. 2022 Mar 21;13(15):4388–96. doi: 10.1039/d1sc06728a (PMC9006924; doi:10.1039/d1sc06728a)
Supplement: SC-013-D1SC06728A-s001 [file SC-013-D1SC06728A-s001.pdf]

Electronic Supplementary Material (ESI) for Chemical Science.
This journal is © The Royal Society of Chemistry 2022

Cucurbiturils mimicked by low polarizability solvents with pre-formed cavities: an empirical model to predict hydrocarbon selectivity

Md Nazimuddin,[a] Héctor Barbero,[a,b] Ramin Rabbani[a] and Eric Masson*[a]

[a] *Department of Chemistry and Biochemistry, Ohio University, Athens, Ohio 45701, United States*

[b] *GIR MIOMeT, IU CINQUIMA/Química Inorgánica, Facultad de Ciencias, Universidad de Valladolid, E47011, Valladolid, Spain.*

e-mail: masson@ohio.edu

Supporting Information

1. Generalities 2
2. Preparation and characterization of probe **P3** and complex CB[8]·**P3**$_2$ 2
4. Hydrocarbon binding assay 6
5. Demonstration of equivalency between equations 6 and 7 11
6. Computational details 11
7. Coordinates of optimized structures 16
8. References 17

1. Generalities

Starting materials were purchased from Sigma-Aldrich (St. Louis, MO), AK Scientific (Union City, CA), Oakwood Chemicals (West Columbia, SC), Fisher Scientific (Hampton, NH), Alfa Aesar (Ward Hill, MA), TCI America (Portland, OR) and Cambridge Isotope Laboratories (Andover, MA). Cucurbit[8]uril (CB[8])[1] was prepared using known procedures. Characterization by nuclear magnetic resonance spectroscopy (NMR) was carried out using a Bruker Ascend 500 spectrometer (Billerica, MA). ^{1}H and ^{13}C NMR chemical shifts are reported in parts per million (ppm) and are referenced to TMS using the residual signal of the solvent as an internal reference. Coupling constants (J) are reported in hertz (Hz). Standard abbreviations used to indicate multiplicity are: s = singlet, d = doublet. Solvent used was deuterated water. Products were also characterized by high-resolution mass spectrometry (HRMS) performed at the COSMIC facility of the Old Dominion University (Norfolk, VA) using a Bruker Daltonics 12 Tesla APEX-Qe FTICR mass spectrometer with an Apollo II Ion Funnel and positive-ion mode electrospray ionization.

2. Preparation and characterization of probe P3 and complex CB[8]·P3$_2$

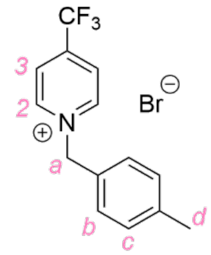

Auxiliary guest P3: 4-(Trifluoromethyl)pyridine (80 mg, 0.54 mmol) and 4-methylbenzyl bromide (0.10 g, 0.54 mmol) were mixed with dry acetonitrile (2.0 mL) and kept at room temperature for 24 h. A white precipitate formed. It was filtered and dried to afford the title compound (0.11 g, 60%). ^{1}H NMR (500 MHz, D_2O): δ 9.20 (d, J = 6.2 Hz, 2H, H^2), 8.44 (d, J = 6.2 Hz, 2H, H^3), 7.41 (d, J = 8.0 Hz, 2H, H^b), 7.37 (d, J = 8.0 Hz, 2H, H^c), 5.91 (s, 2H, H^a), 2.38 (s, 3H, H^d). ^{13}C NMR (126 MHz, D_2O): δ 146.21, 146.13, 140.94, 130.20, 129.43, 128.77, 125.29, 125.27, 122.02, 119.83, 65.20, 20.28. ^{19}F NMR (471 MHz, D_2O): δ -65.49. HRMS (ESI-FTICR): m/z = 252.099242 [M]$^+$(calcd. 252.099460 for $C_{14}H_{13}F_3N$).

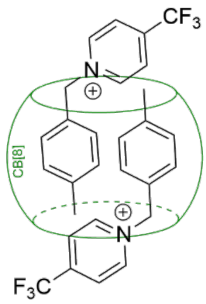

Assembly CB[8]·P3$_2$. Guest **P3** (3.3 mg, 10 µmol) was mixed with D_2O (5.0 mL). CB[8] (10 mg, 5.0 µmol) was added subsequently, and the resulting mixture was sonicated thoroughly. The stock solution was stored at 4 ºC for further use. ^{1}H NMR (500 MHz, D_2O): δ 9.23 (d, J = 3.9 Hz, 4H, H^2), 8.55 (s, 4H, H^3), 6.65 (d, J = 5.9 Hz 4H, H^b), 5.99 (s, 4H, H^c), 5.82 (d, J = 15.3 Hz, 16H, H$^{CB[8]}$), 5.57 (s, 4H, H^a), 5.54 (s, 16H, H$^{CB[8]}$), 4.24 (d, J = 15.3 Hz, 16H, H$^{CB[8]}$),

1.05 (s, 6H, H^{d}). ^{13}C NMR (126 MHz, D_2O): δ 156.66, 146.32, 138.90, 128.82, 128.56, 127.51, 125.45, 122.19, 120.01, 72.06, 65.38, 53.65, 20.44. ^{19}F NMR (471 MHz, D_2O): δ -65.19. HRMS (ESI): m/z = 916.29547 $[M]^{2+}$ (calcd. 916.29631 for $C_{76}H_{74}N_{34}O_{16}F_6$).

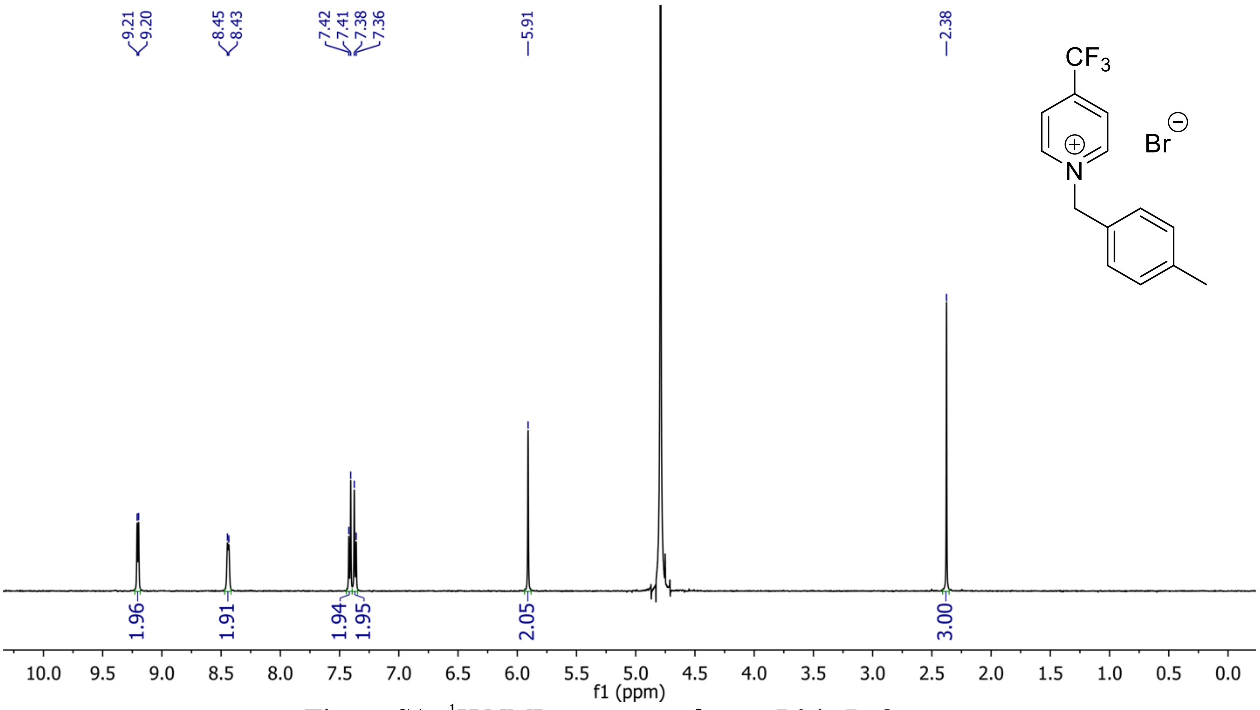

Figure S1. ^{1}H NMR spectrum of guest **P3** in D_2O.

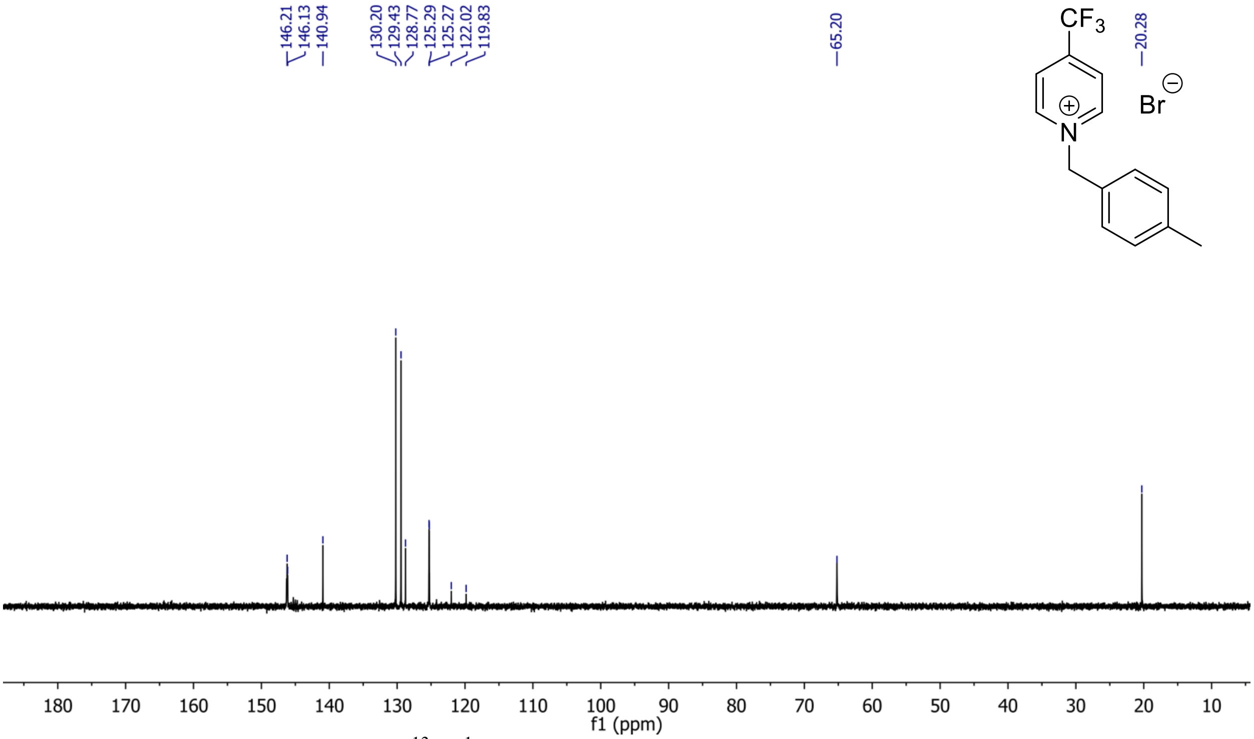

Figure S2. $^{13}C\{^{1}H\}$ NMR spectrum of guest **P3** in D_2O.

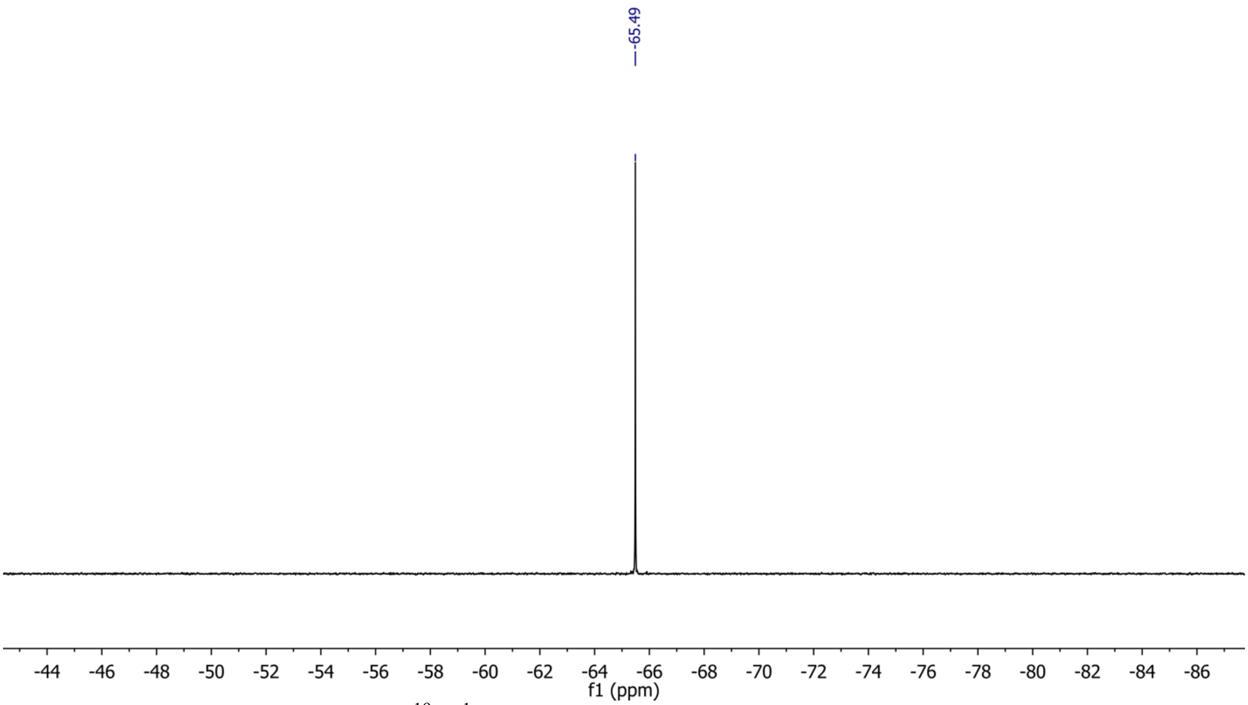

Figure S3. $^{19}F\{^{1}H\}$ NMR spectrum of guest **P3** in D_2O.

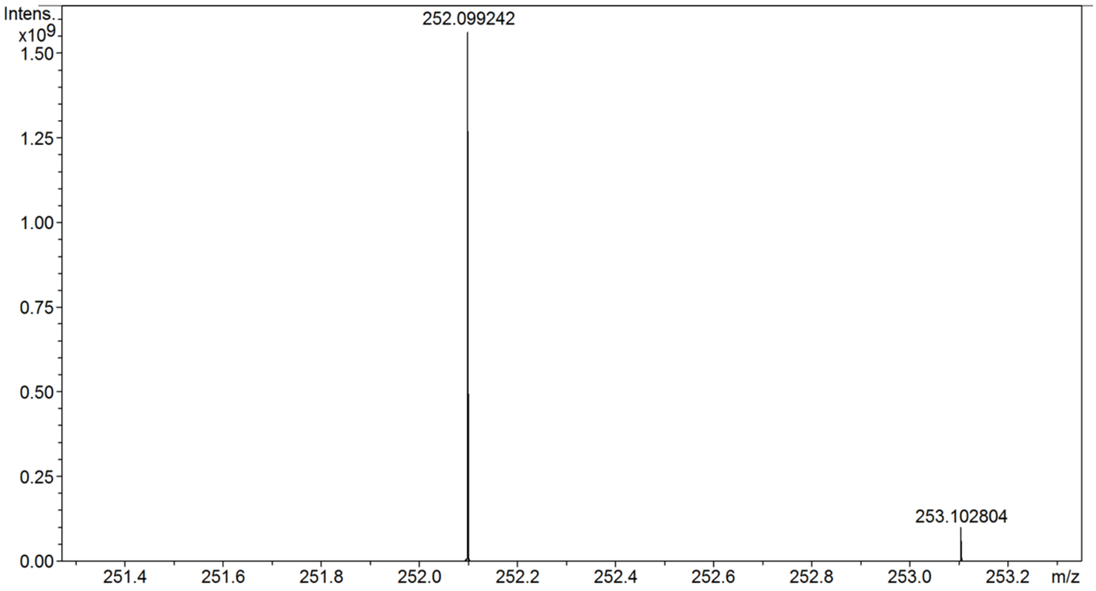

Figure S4. MS spectrum of guest **P3**.

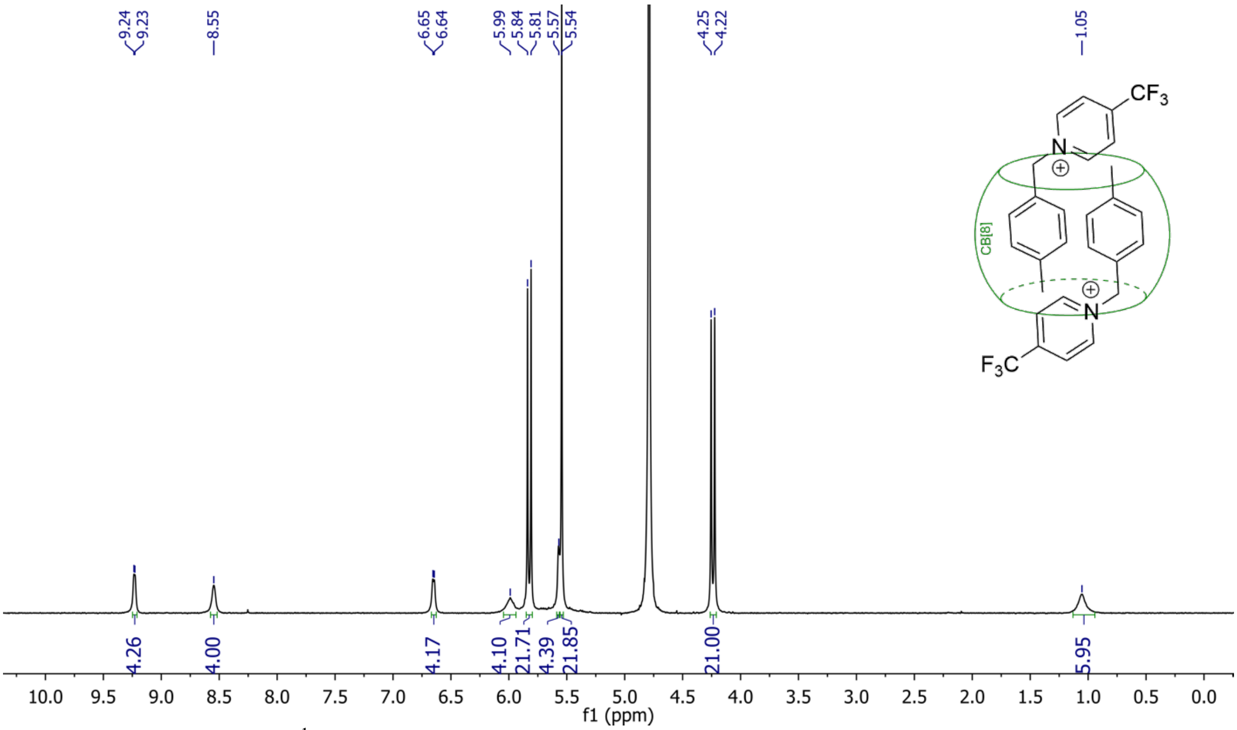

Figure S5. ^{1}H NMR spectrum of homoternary complex CB[8]·**P3**$_2$ in D_2O.

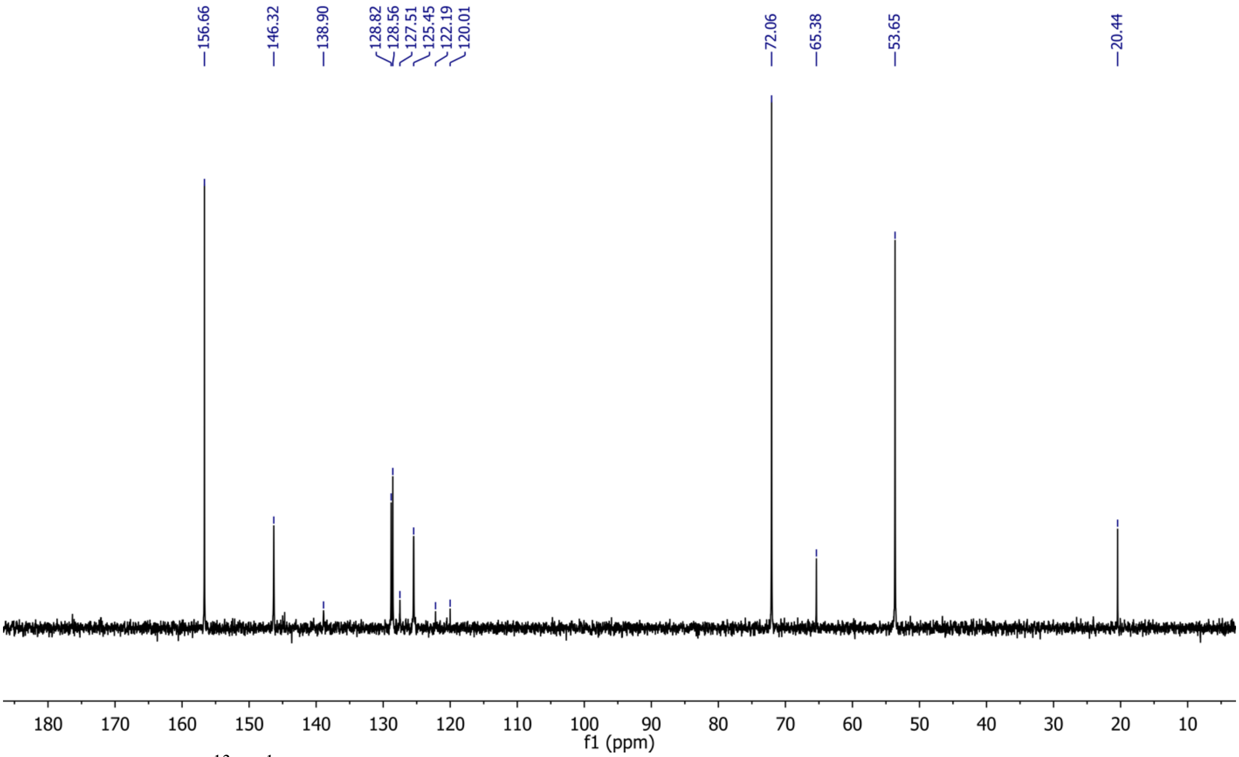

Figure S6. $^{13}C\{^{1}H\}$ NMR spectrum of homoternary complex complex CB[8]·**P3**$_2$ in D_2O.

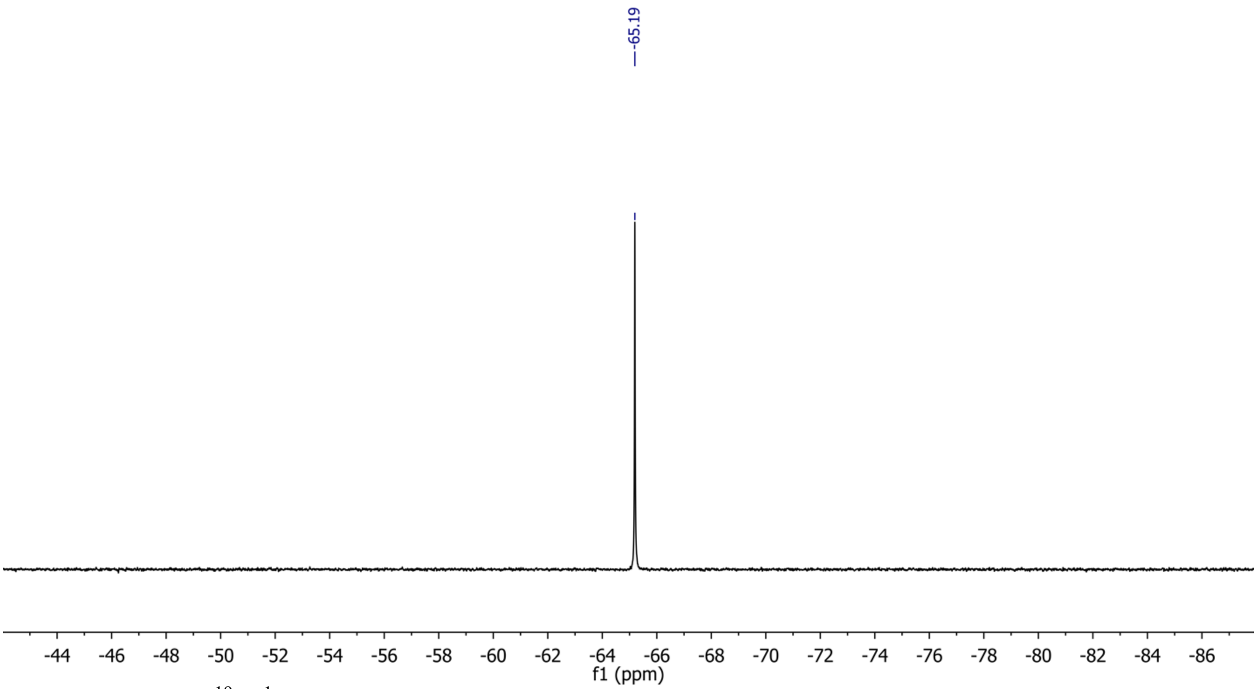

Figure S7. $^{19}F\{^{1}H\}$ NMR spectrum of homoternary complex complex CB[8]·**P3**$_2$ in D_2O.

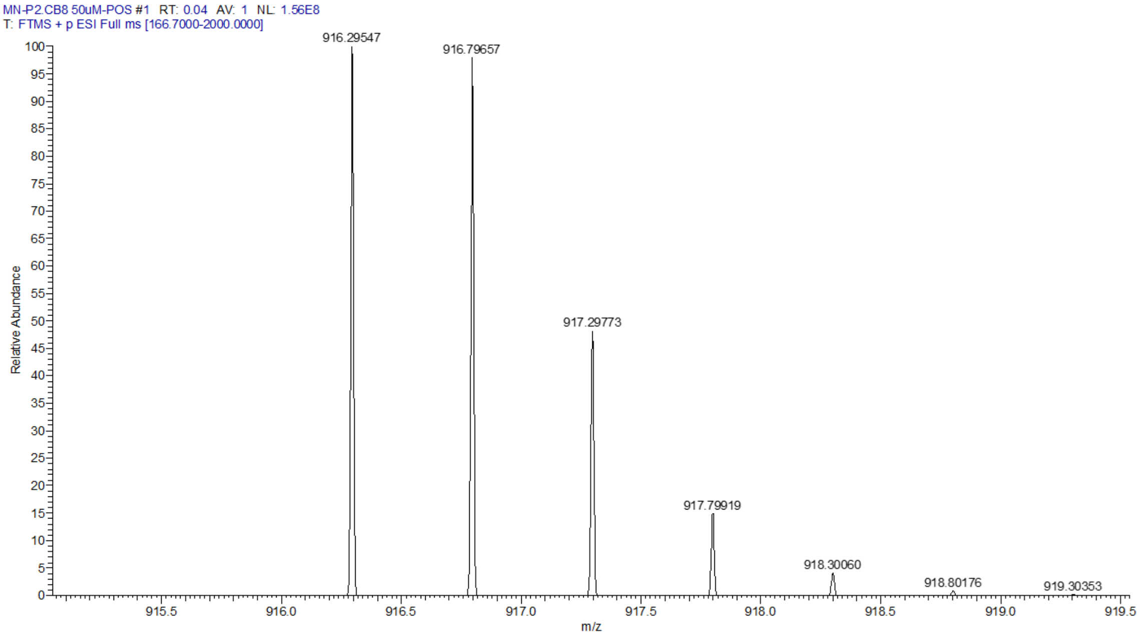

Figure S8. MS spectrum of homoternary complex CB[8]·**P3**$_2$.

4. Hydrocarbon binding assay

A solution of auxiliary guest **P3** (10 mg, 30 µmol) in deuterium oxide (15 mL) was prepared. CB[8] (40 mg, 30 µmol) was added, and the mixture was sonicated for 10 min at 25 ºC to afford a milky emulsion. Samples (0.40 mL) were withdrawn immediately after sonication and transferred to NMR tubes equipped with an insert containing a standard for quantification (2,2,2-trifluoroethylammonium chloride in deuterium oxide; 0.10 mL, 10 mM). A reference

hydrocarbon (0.10 mL) was added to the NMR tube, aliquots of the second hydrocarbon (2.5, 5.0, 7.5, 10 μL etc.) were added, and the NMR tube was sonicated at 25 °C for 10 min after each addition. $^{19}F\{^1H\}$ NMR spectra were recorded after each hydrocarbon addition (see Figures S9 to S12). All spectra were recorded on the day of sample preparation. We note that sonication times and bath temperatures did not significantly affect binding affinities.

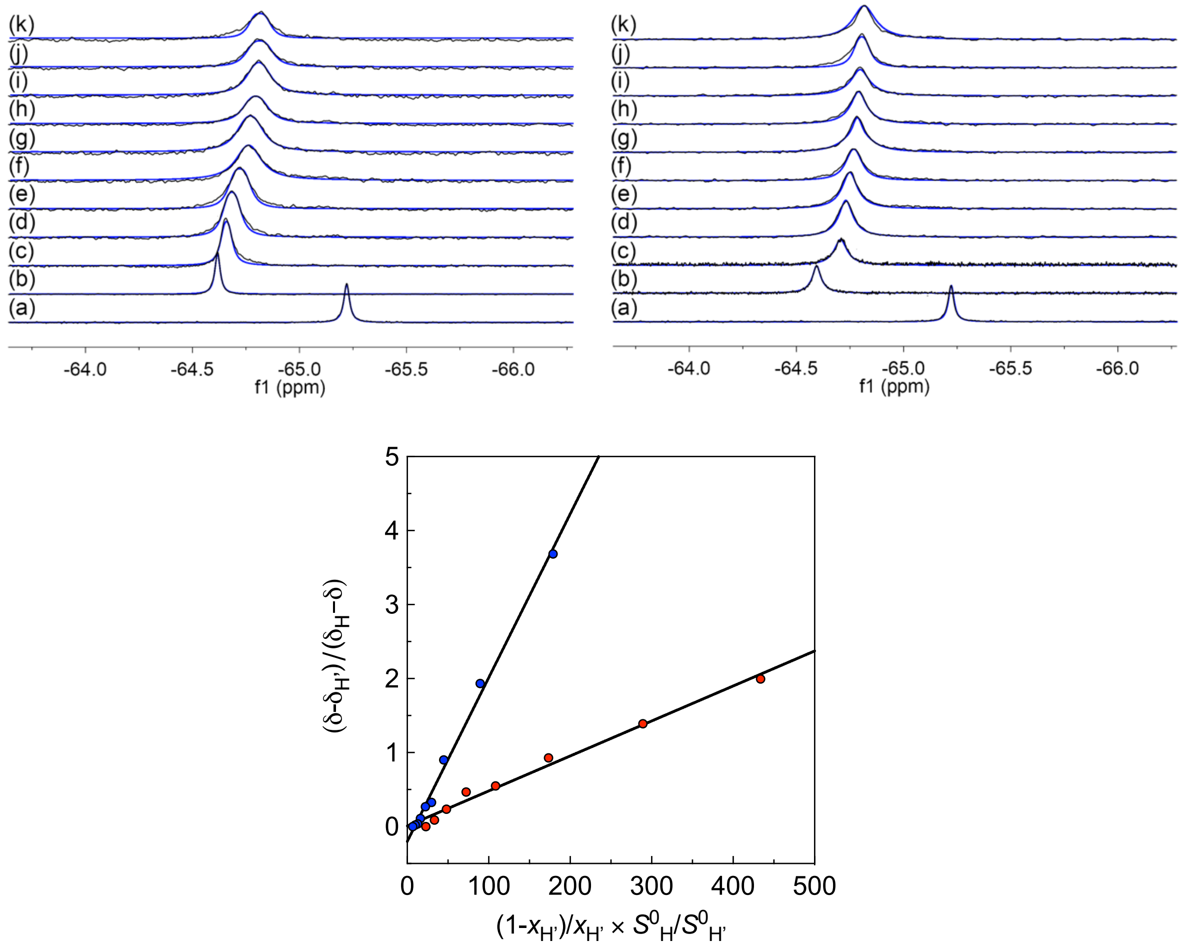

Figure S9. *Top left* – $^{19}F\{^1H\}$ spectra of (a) complex CB[8]·**P3**$_2$ in deuterium oxide (0.40 mL), (b) complex CB[8]·**P3**·cyclopentane after addition of cyclopentane (0.10 mL) and sonication; mixtures of complexes CB[8]·**P3**·cyclopentane and CB[8]·**P3**·cycloheptene after addition of (c) 2.5, (d) 5.0, (e) 10, (f) 15, (g) 20, (h) 28, (i) 35, (j) 45, and (k) 65 μL cycloheptene. *Top right* – $^{19}F\{^1H\}$ spectra of (a) complex CB[8]·**P3**$_2$ in deuterium oxide (0.40 mL), (b) complex CB[8]·**P3**·cyclopentene after addition of cyclopentene (0.10 mL) and sonication; mixtures of complexes CB[8]·**P3**·cyclopentene and CB[8]·**P3**·cycloheptene after addition of (c) 2.5, (d) 5.0, (e) 7.5, (f) 13, (g) 20, (h) 30, (i) 45, (j) 65, and (k) 95 μL cycloheptene. *Bottom* – plot of $(\delta - \delta_{H'})/(\delta_H - \delta)$ as a function of $(1 - x_{H'})S^0_H/(x_{H'}S^0_{H'})$, where δ_H are the chemical shifts of complexes CB[8]·**P3**·cyclopentane (blue series) or CB[8]·**P3**·cyclopentene (red series), $\delta_{H'}$ the chemical shifts of complex CB[8]·**P3**·cycloheptene, and δ chemical shifts of mixtures; S^0 and x are hydrocarbon solubilities and molar fractions in the mixture; see manuscript for details. Chemical shifts measured at the maximum of the Gaussian function used to fit the spectra. Experimental and fitted spectra in black and blue, respectively.

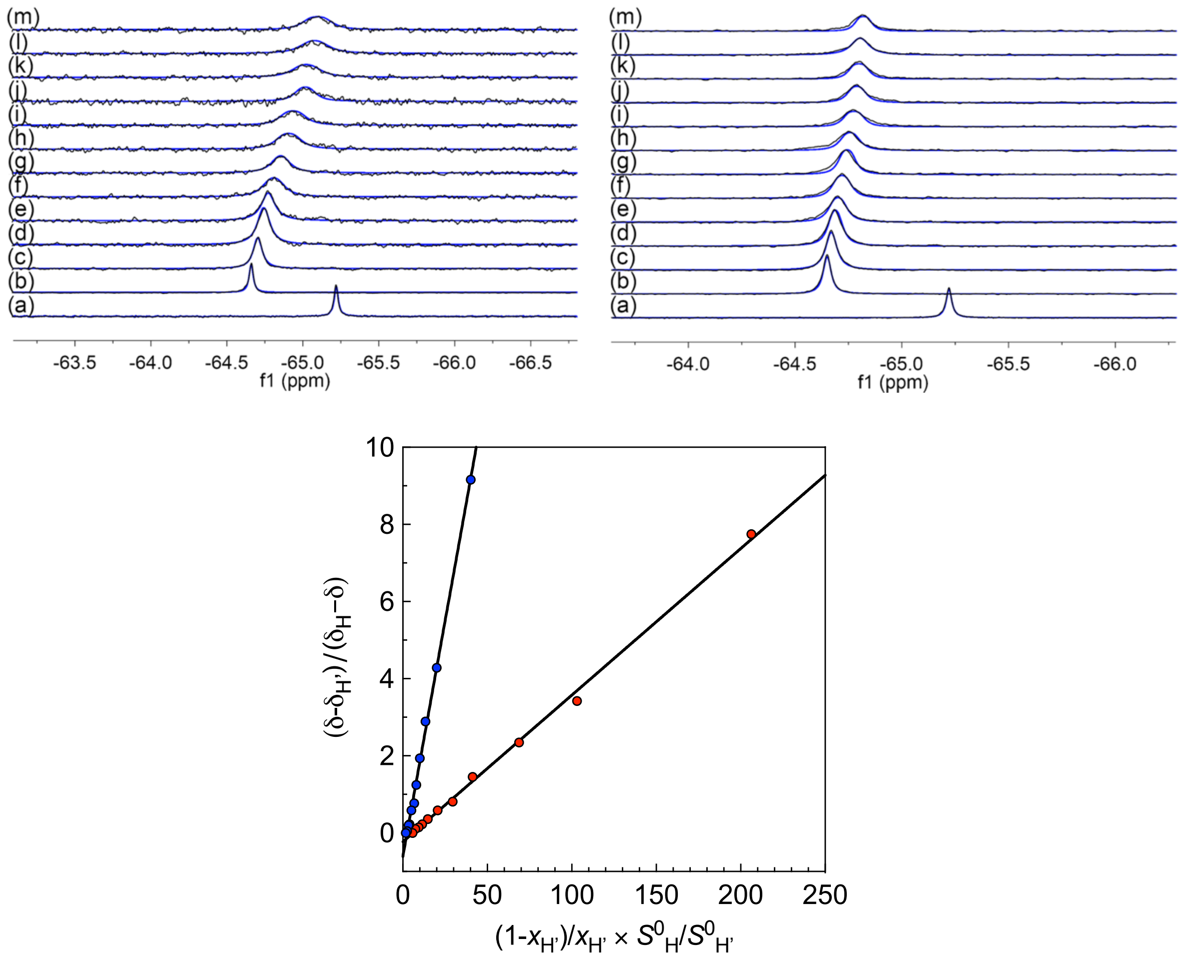

Figure S10. *Top left* – ^{19}F{^{1}H} spectra of (a) complex CB[8]·**P3**$_2$ in deuterium oxide (0.40 mL), (b) complex CB[8]·**P3**·cyclohexane after addition of cyclohexane (0.10 mL) and sonication; mixtures of complexes CB[8]·**P3**·cyclohexane and CB[8]·**P3**·cycloheptene after addition of (c) 2.5, (d) 5.0, (e) 7.5, (f) 10, (g) 12.5, (h) 15, (i) 20, (j) 25, (k) 30, (l) 40, (m) 60 µL cycloheptene. *Top right* – ^{19}F{^{1}H} spectra of (a) complex CB[8]·**P3**$_2$ in deuterium oxide (0.40 mL), (b) complex CB[8]·**P3**·cyclohexene after addition of cyclohexene (0.10 mL) and sonication; mixtures of complexes CB[8]·**P3**·cyclohexene and CB[8]·**P3**·cycloheptene after addition of (c) 2.5, (d) 5.0, (e) 7.5, (f) 13, (g) 18, (h) 25, (i) 35, (j) 45, (k) 55, (l) 70, (m) 90 µL cycloheptene. *Bottom* – plot of $(\delta - \delta_{H'})/(\delta_H - \delta)$ as a function of $(1 - x_{H'})S^0_H/(x_{H'}S^0_{H'})$, where δ_H are the chemical shifts of complexes CB[8]·**P3**·cyclohexane (blue series) or CB[8]·**P3**·cyclohexene (red series), $\delta_{H'}$ the chemical shifts of complex CB[8]·**P3**·cycloheptene, and δ chemical shifts of mixtures; S^0 and x are hydrocarbon solubilities and molar fractions in the mixture; see manuscript for details. Chemical shifts measured at the maximum of the Gaussian function used to fit the spectra. Experimental and fitted spectra in black and blue, respectively.

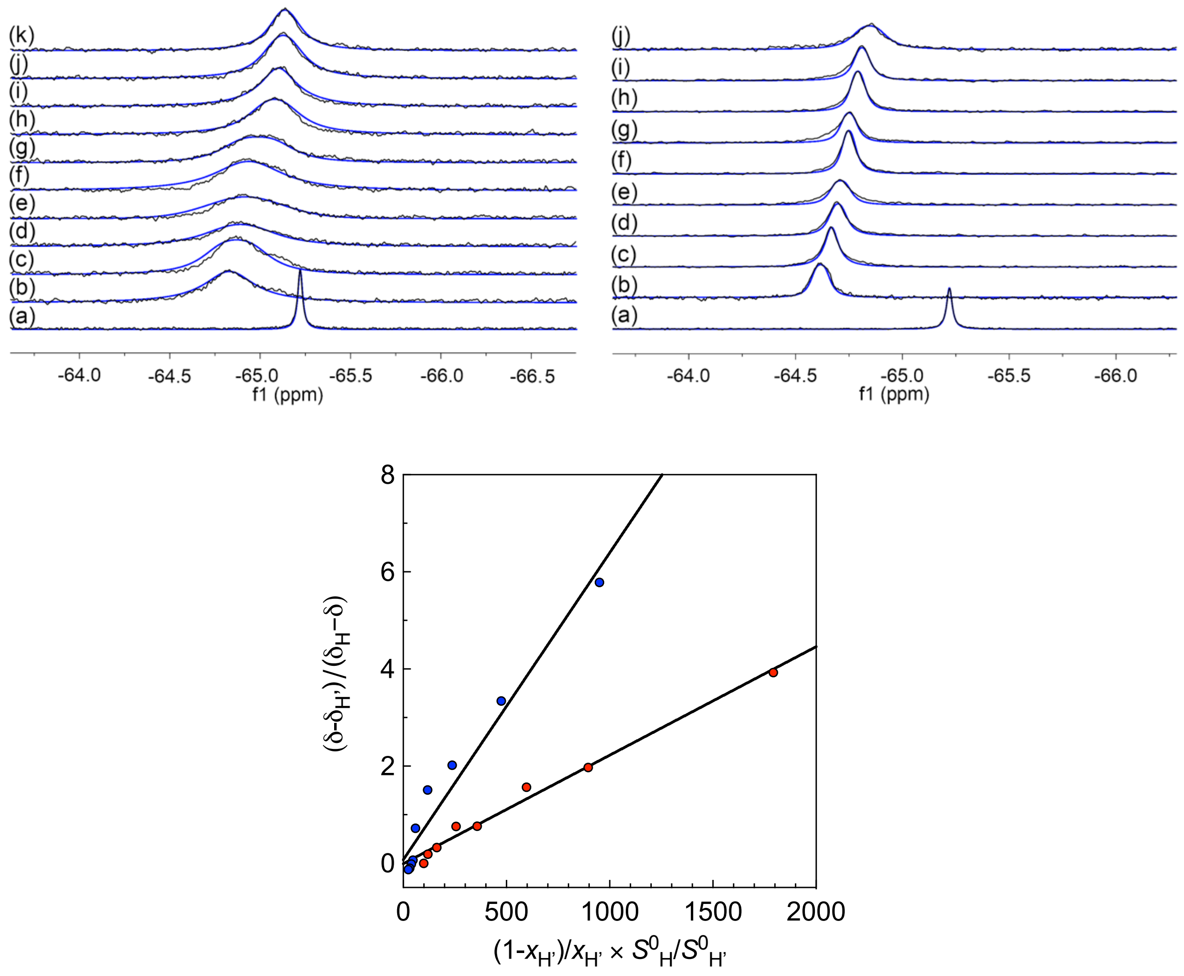

Figure S11. *Top left* – $^{19}F\{^{1}H\}$ spectra of (a) complex CB[8]·**P3**$_2$ in deuterium oxide (0.40 mL), (b) complex CB[8]·**P3**·1,3-cyclohexadiene after addition of 1,3-cyclohexadiene (0.10 mL) and sonication; mixtures of complexes CB[8]·**P3**·1,3-cyclohexadiene and CB[8]·**P3**·cycloheptene after addition of (c) 2.5, (d) 5.0, (e) 10, (f) 20, (g) 40, (h) 50, (i) 60, (j) 75, and (k) 95 μL cycloheptene. *Top right* – $^{19}F\{^{1}H\}$ spectra of (a) complex CB[8]·**P3**$_2$ in deuterium oxide (0.40 mL), (b) complex CB[8]·**P3**·benzene after addition of benzene (0.10 mL) and sonication; mixtures of complexes CB[8]·**P3**·benzene and CB[8]·**P3**·cycloheptene after addition of (c) 2.5, (d) 5.0, (e) 7.5, (f) 13, (g) 18, (h) 18, (i) 38, (j) 45 μL cycloheptene. *Bottom* – plot of $(\delta - \delta_{H'})/(\delta_H - \delta)$ as a function of $(1 - x_{H'})S^0_H/(x_{H'}S^0_{H'})$, where δ_H are the chemical shifts of complexes CB[8]·**P3**·1,3-cyclohexadiene (blue series) or CB[8]·**P3**·benzene (red series), $\delta_{H'}$ the chemical shifts of complex CB[8]·**P3**·cycloheptene, and δ chemical shifts of mixtures; S^0 and x are hydrocarbon solubilities and molar fractions in the mixture; see manuscript for details. Chemical shifts measured at the maximum of the Gaussian function used to fit the spectra. Experimental and fitted spectra in black and blue, respectively.

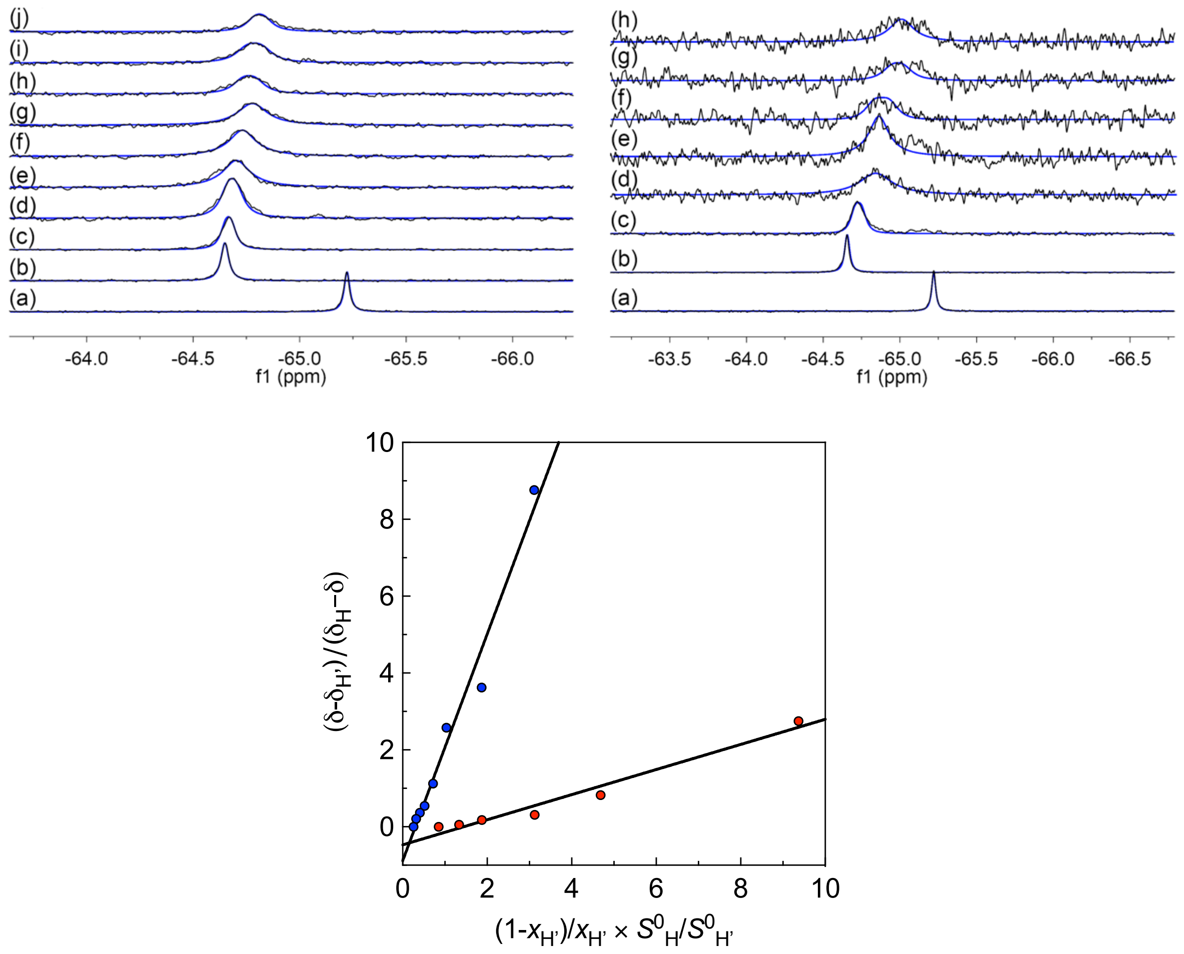

Figure S12. *Top left* – ^{19}F{^{1}H} spectra of (a) complex CB[8]·**P3**$_2$ in deuterium oxide (0.40 mL), (b) complex CB[8]·**P3**·cyclohexene after addition of cyclohexene (0.10 mL) and sonication; mixtures of complexes CB[8]·**P3**·cyclohexene and CB[8]·**P3**·1,4-cyclohexadiene after addition of (c) 7.5, (d) 13, (e) 23, (f) 33, (g) 45, (h) 58, (i) 73, and (j) 90 μL 1,4-cyclohexadiene. *Top right* – ^{19}F{^{1}H} spectra of (a) complex CB[8]·**P3**$_2$ in deuterium oxide (0.40 mL), (b) complex CB[8]·**P3**·cyclohexane after addition of cyclohexane (0.10 mL) and sonication; mixtures of complexes CB[8]·**P3**·cyclohexane and CB[8]·**P3**·cyclooctatetraene after addition of (c) 2.5, (d) 5.0, (e) 7.5, (f) 13, (g) 18, (h) 28 μL cyclooctatetraene. *Bottom* – plot of $(\delta - \delta_{H'})/(\delta_H - \delta)$ as a function of $(1 - x_{H'})S^0_H/(x_{H'}S^0_{H'})$, where δ_H are the chemical shifts of complexes CB[8]·**P3**·cyclohexene (blue series) or CB[8]·**P3**·cyclohexane (red series), $\delta_{H'}$ the chemical shifts of complex CB[8]·**P3**·1,4-cyclohexadiene (blue series) or CB[8]·**P3**·cyclooctatetraene (red series), and δ chemical shifts of mixtures; S^0 and x are hydrocarbon solubilities and molar fractions in the mixture; see manuscript for details. Chemical shifts measured at the maximum of the Gaussian function used to fit the spectra. Experimental and fitted spectra in black and blue, respectively.

Table S1. Relative binding affinities of hydrocarbons to assemblies CB[8]·**P3**, and their corresponding free energies of transfer from the gas phase to the cavities of the binary complexes, by forcing (or not) the regression lines of the ^{19}F NMR competitive experiments through origin.

Hydrocarbon	not through origin				through origin			
	$K_{\mathrm{aq}\rightarrow\mathrm{CB}}^{\mathrm{rel,benzene}}$		$\Delta G_{\mathrm{gas}\rightarrow\mathrm{CB}}^{\mathrm{rel,benzene}}$		$K_{\mathrm{aq}\rightarrow\mathrm{CB}}^{\mathrm{rel,benzene}}$		$\Delta G_{\mathrm{gas}\rightarrow\mathrm{CB}}^{\mathrm{rel,benzene}}$	
cyclopentane	9.9	(± 0.3)	+0.66	(± 0.02)	9.1	(± 0.4)	+0.71	(± 0.03)
cyclopentene	2.1	(± 0.1)	+0.86	(± 0.03)	2.1	(± 0.1)	+0.86	(± 0.02)
cyclohexane	110	(± 2)	–0.82	(± 0.01)	97	(± 4)	–0.74	(± 0.03)
cyclohexene	17.0	(± 0.3)	–0.58	(± 0.01)	16.1	(± 0.4)	–0.54	(± 0.01)
1,3-cyclohexadiene	2.8	(± 0.2)	–0.20	(± 0.05)	2.9	(± 0.2)	–0.21	(± 0.04)
1,4-cyclohexadiene	5.8	(± 0.4)	–0.76	(± 0.04)	6.5	(± 0.6)	–0.84	(± 0.05)
benzene	1.00	(± 0.05)	0.00	(± 0.03)	1.0	(± 0.03)	0.00	(± 0.02)
cycloheptene	448		–2.71		449		–2.71	
cyclooctatetraene	334	(± 32)	–4.40	(± 0.06)	391	(± 56)	–4.49	(± 0.09)

5. Demonstration of equivalency between equations 6 and 7

$$\Delta G_{\mathrm{solv}}^{i} = -RT \ln \frac{S_i \cdot RT}{P_{\mathrm{vap},i}} \qquad (6)$$

$$\Delta G_{\mathrm{solv}}^{i} = -RT \ln \frac{S_i \cdot P^0}{P_{\mathrm{vap},i}} - 1.90\ \mathrm{kcal/mol} \qquad (7)$$

Nau reminded the reader[2] that, in the molar reference framework, 1 M in ideal gas concentration corresponds to a pressure of 24.5 atm, i.e. p [Pa] = RT. As the 1.90 kcal/mol correction term is intended to be RT ln 24.5, equation 7 becomes:

$$\Delta G_{\mathrm{solv}}^{i} = -RT \ln \frac{S_i \cdot P^0 \cdot 24.5}{P_{\mathrm{vap},i}}$$

With P^0 being 101325 Pa and converting atm into Pa, $P^0 \cdot 24.5$ is thus equal to RT, thereby affording back equation 6.

6. Computational details

All calculations were carried out with Gaussian 16 Rev. C.01[3] (g16), the Turbomole suite of programs (TM; version 7.2.1)[4–8] or GFN2-XTB[9–11] on the OSC Owens and Pitzer Clusters of the Ohio Supercomputer Center in Columbus, OH (23,392-core Dell Intel Xeon E5-2680 v4 and 10,240-core Dell Intel Gold 6148 machines, respectively).

Hydrocarbon volumes, delimited by a 0.002 electron/Bohr^3 isodensity surface, were calculated at the semi-empirical PM6 level with g16. Volumes of noble gases were calculated at the TPSS-D3(BJ)/def2-TZVP level.

Complexes CB[8]·**P3**·hydrocarbon were optimized at the semi-empirical GFN2-xTB level[9–11] with the alpb solvation model[12] in the molar reference state.

Methane and putative methane dimers and trimers, as well as the methane/water heterodimer were optimized with g16[3] at the TPSS-D3(BJ)/def2-TZVP level.[13–17] Vibrational analysis was carried out at the same level of theory, and free energy terms were extracted with the GoodVibes 3.0 program[18] with quasi-harmonic enthalpic and entropic corrections[19–21] for frequencies below 100 cm^{-1}.

Free energies of solvation of hydrocarbons and noble gases were calculated using the procedure described by Klamt and coworkers using TM.[22] All hydrocarbons were optimized (a) in the gas phase, at the BP86/def-TZVP level of theory,[14,15,23–25] and (b) in solution at the same level of theory using the COSMO solvation model.[26] Energies were then refined in single-point calculations at the BP86/def2-TZVPD level. The output from these single-point calculations in the gas phase and in solution was then treated with COSMO*therm* X18[27] and the BP_TZVPD_FINE_18 parametrization model to extract free Gibbs energies of solvation.

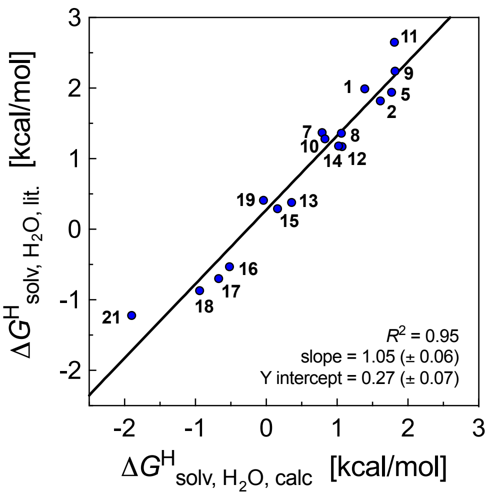

Figure S13. Comparison of calculated free energies of solvation of hydrocarbons in water with experimental data.[2,28–32] See manuscript for hydrocarbon numbering.

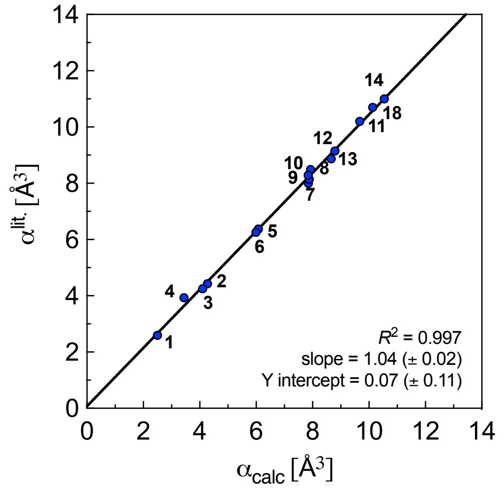

Figure S14. Comparison of hydrocarbon static polarizabilities calculated at the pbe0/aug-cc-pVTZ level[33–35] with experimental data.[2,28–32] See manuscript for hydrocarbon numbering.

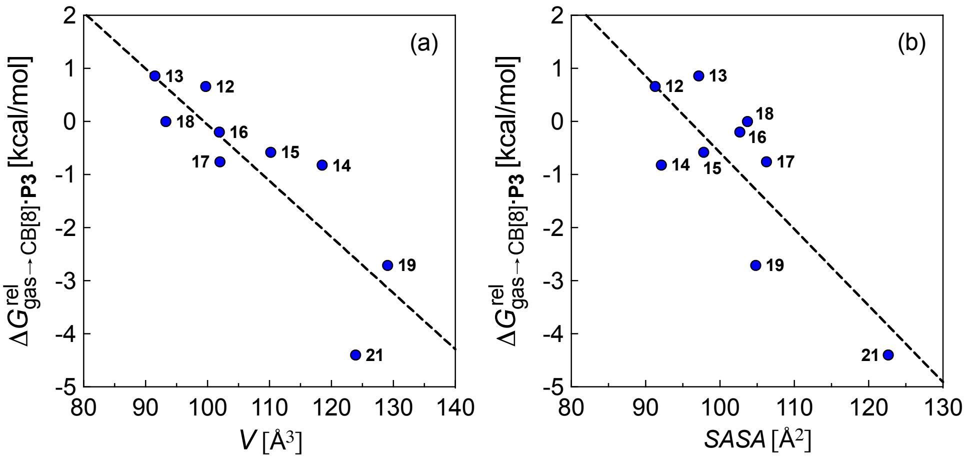

Figure S15. Relative free energies of transfer of hydrocarbons from the gas phase to the cavity of assembly CB[8]·**P3** $\Delta G^{rel}_{gas \rightarrow CB[8] \cdot \mathbf{P3}}$ as a function of (a) hydrocarbon volume, and (b) hydrocarbon solvent-accessible surface area. See manuscript for hydrocarbon numbering.

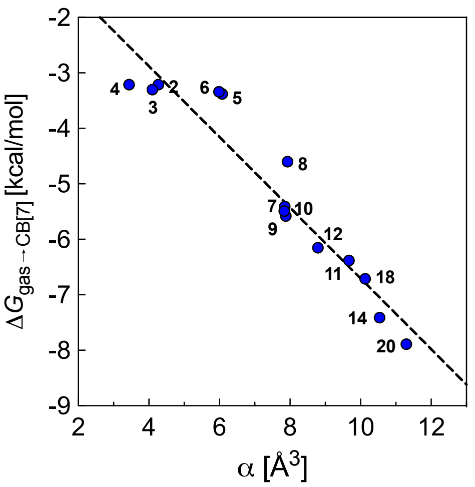

Figure S16. Free energies of binding $\Delta G_{gas \rightarrow CB[7]}$ as a function of hydrocarbon polarizability. See manuscript for hydrocarbon numbering.

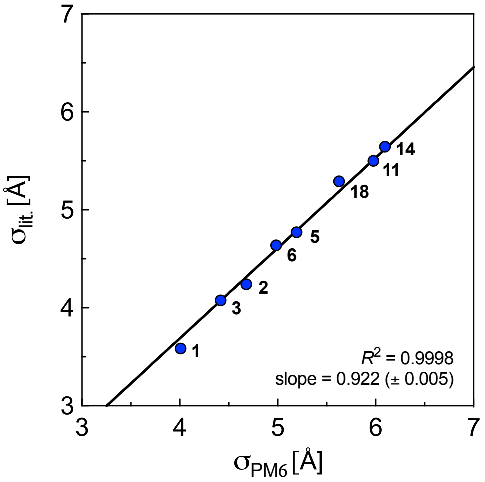

Figure S17. Tabulated diameters[36] of hydrocarbons considered as hard spheres, as a function of diameters calculated from the volumes of their PM6 optimized structures delimited by a 0.002 electron/Bohr^3 isodensity surface. See manuscript for hydrocarbon numbering.

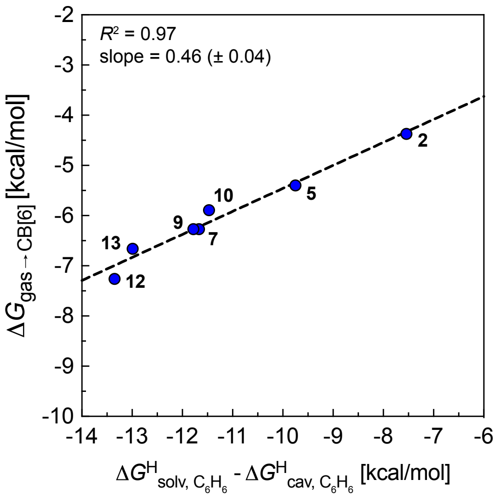

Figure S18. Free energies of transfer of hydrocarbons from the gas phase to the cavity of CB[6] $\Delta G_{\mathrm{gas}\rightarrow\mathrm{CB[6]}}$ as a function of the energy released upon introduction of the hydrocarbon into a pre-formed cavity in benzene ($\Delta G^{\mathrm{H}}_{\mathrm{solv,C_6H_6}} - \Delta G^{\mathrm{H}}_{\mathrm{cav,C_6H_6}}$). See manuscript for hydrocarbon numbering.

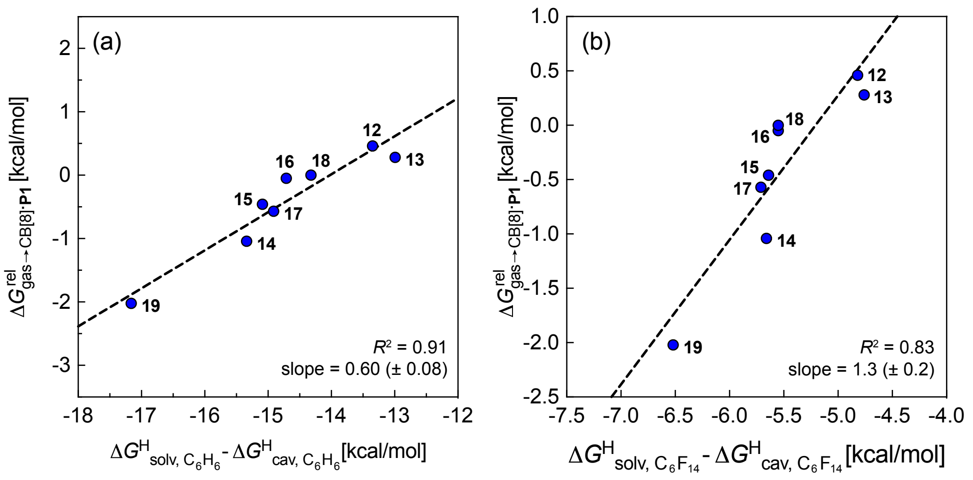

Figure S19. Free energies of transfer of hydrocarbons from the gas phase to the cavity of CB[8]·**P1** relative to benzene $\Delta G^{\mathrm{rel}}_{\mathrm{gas}\rightarrow\mathrm{CB[8]\cdot\mathbf{P1}}}$ as a function of the energy released upon introduction of the hydrocarbon into a pre-formed cavity in (a) benzene ($\Delta G^{\mathrm{H}}_{\mathrm{solv,C_6H_6}} - \Delta G^{\mathrm{H}}_{\mathrm{cav,C_6H_6}}$) and (b) perfluorohexane ($\Delta G^{\mathrm{H}}_{\mathrm{solv,C_6F_{14}}} - \Delta G^{\mathrm{H}}_{\mathrm{cav,C_6F_{14}}}$). See manuscript for hydrocarbon numbering.

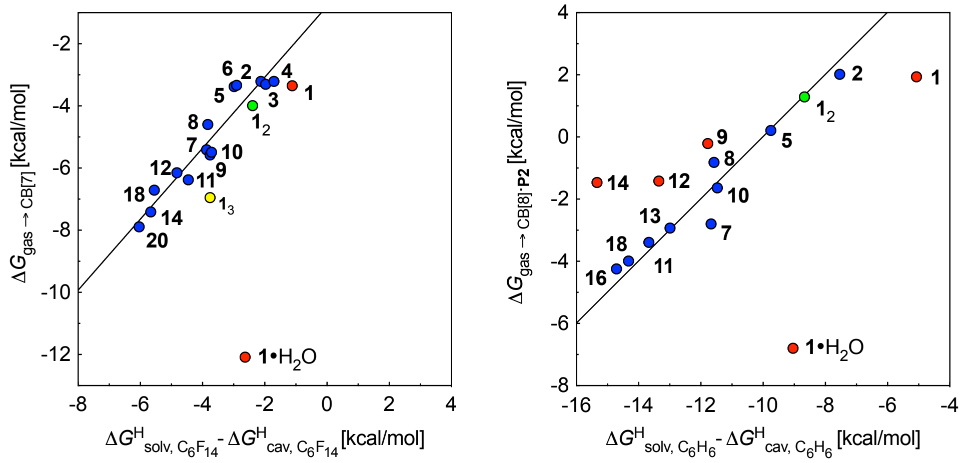

Figure S20. Free energies of transfer of hydrocarbons from the gas phase to the cavity of CB[7] ($\Delta G_{\text{gas}\rightarrow\text{CB[7]}}$) and to assembly CB[8]·**P2** ($\Delta G_{\text{gas}\rightarrow\text{CB[8]}\cdot\mathbf{P2}}$) as a function of the energy released upon introduction of the hydrocarbon into a pre-formed cavity in perfluorohexane ($\Delta G^{\text{H}}_{\text{solv},\text{C}_6\text{F}_{14}} - \Delta G^{\text{H}}_{\text{cav},\text{C}_6\text{F}_{14}}$) and benzene ($\Delta G^{\text{H}}_{\text{solv},\text{C}_6\text{H}_6} - \Delta G^{\text{H}}_{\text{cav},\text{C}_6\text{H}_6}$), respectively. See Table 2 for hydrocarbon numbering. Outliers **1** and **1**·H_2O are highlighted in red.

Table S2. Physicochemical and thermodynamic properties of noble gases (He – Xe), methane (1) and ethane (2) as well as their CB[5] complexes.

Guest	V [a]	σ' [b]	α [c]	$\Delta G^{\text{He-Xe,H}}_{\text{solv}}$ [d]			$\Delta G^{\text{He-Xe,H}}_{\text{cav}}$ [e]		$\Delta G_{\text{gas}\rightarrow\text{CB[5]}}$ [f]
				H_2O	C_6H_6	C_6F_{14}	C_6H_6	C_6F_{14}	
He	7.5	2.24	0.22	2.90	2.54	2.51	0.39	1.74	0.30
Ne	11.3	2.57	0.37	2.92	2.33	2.33	–0.31	1.43	0.42
Ar	25.1	3.35	1.63	2.24	0.93	1.20	–3.16	–0.10	–1.26
Kr	31.3	3.61	2.52	1.91	0.53	0.89	–4.15	–0.56	–2.69
Xe	42.6	4.00	4.01 [g]	1.77	–0.19	0.31	–5.86	–1.40	–3.63
methane (**1**)	33.7	3.70	2.50	1.39	–0.18	0.39	4.89	1.51	–3.01
ethane (**2**)	53.6	4.32	4.27	1.61	–0.97	–0.18	6.58	1.93	–0.29

[a] Guest volume calculated at the TPSS-D3(BJ)/def2-TZVP level and delimited by a 0.002 electron/Bohr^3 isodensity surface; in Å^3. [b] Effective hard sphere diameter obtained from equation 12 (see narrative); in Å. [c] Static polarizability calculated at the pbe0/aug–cc–pVTZ level; in Å^3. [d] Free energies of solvation in water, benzene and perfluorohexane, calculated with the COSMO-RS solvation model and the Cosmotherm software; in kcal/mol. [e] Cavitation energies in benzene and perfluorohexane, obtained from equations 10 – 12 (see narrative); in kcal/mol. [f] Free energies of transfer from the gas phase (molar reference state) to the cavity of CB[5] in aqueous solution; in kcal/mol. [g] from ref. 37.

7. Coordinates of optimized structures

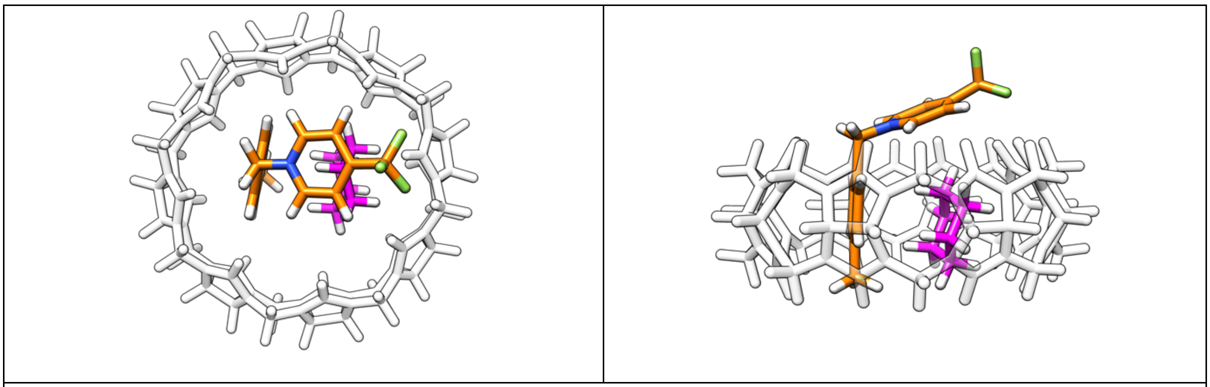

CB[8]·**P3**·cyclohexane

C	1.32460	0.99700	0.77530	O	-1.68730	-3.34760	5.88800	H	-3.79270	-4.30150	-2.03870
C	0.07700	1.78070	3.15770	N	-3.98590	-4.74060	-0.06000	N	-3.39410	5.28650	0.17780
C	1.56030	0.30200	1.95470	C	-4.73510	-5.21290	1.07660	C	-2.67410	5.78690	-0.97080
C	0.47490	2.09530	0.79730	C	-3.64710	-5.86000	1.97160	C	-1.34310	6.30230	-0.36480
C	-0.13590	2.48400	1.97700	N	-2.45070	-5.72690	1.18730	N	-1.49860	6.04890	1.04670
C	0.94730	0.69420	3.13120	C	-2.64540	-5.00840	0.03800	C	-2.68880	5.43680	1.34490
H	2.22580	-0.54790	1.94780	H	-5.54010	-5.88560	0.75050	H	-3.27600	6.53700	-1.50030
H	0.28430	2.64240	-0.11310	H	-3.81640	-6.91320	2.23410	H	-1.12200	7.36340	-0.53910
H	-0.80680	3.33240	1.99120	N	-5.27310	-4.16170	1.90530	N	-2.24630	4.75280	-1.87460
H	1.11940	0.14910	4.05010	N	-3.67020	-5.04210	3.16290	N	-0.35020	5.46870	-0.99030
C	1.99770	0.57750	-0.51280	C	-4.64230	-4.07710	3.12040	C	-0.90060	4.51570	-1.80420
H	2.88260	-0.01300	-0.26970	O	-1.79760	-4.73410	-0.78090	O	-3.07590	5.14040	2.45560
H	2.30760	1.47680	-1.04960	O	-4.94700	-3.33330	4.02800	O	-0.29190	3.65780	-2.40600
N	1.10610	-0.20530	-1.35490	C	2.88870	-4.80730	3.34840	C	1.05530	5.76410	-0.98740
C	-0.48820	-1.70390	-3.00980	H	3.38550	-5.58220	3.94560	H	1.50410	5.18490	-1.80250
C	0.37860	0.40170	-2.29520	H	3.22600	-4.83730	2.30610	H	1.18980	6.84030	-1.15080
C	1.00910	-1.52440	-1.16330	C	1.11460	-3.79200	6.33460	C	-0.65990	6.64910	2.04900
C	0.21590	-2.31380	-1.97430	H	0.44260	-3.24730	7.00780	H	-1.20580	6.59380	2.99810
C	-0.42900	-0.32190	-3.15350	H	1.63640	-4.59650	6.87010	H	-0.46950	7.69330	1.76900
H	0.45530	1.47740	-2.39160	N	-5.57900	-0.99200	-2.11290	C	2.94770	2.44700	5.53080
H	1.60110	-1.96570	-0.37320	C	-6.74930	-1.27190	-1.32630	N	3.43650	3.38570	4.66390
H	0.14850	-3.37680	-1.79890	C	-6.45410	-2.67720	-0.73990	C	4.65630	2.96230	4.01930
H	-1.00670	0.19130	-3.90560	N	-5.13930	-2.98010	-1.24570	C	4.89440	1.54430	4.59990
C	-1.18790	-2.54570	-4.04690	C	-4.61230	-1.95130	-1.98020	N	3.77520	1.35750	5.49180
F	-0.27310	-2.98760	-4.95350	H	-7.65420	-1.19690	-1.94360	H	5.45570	3.69060	4.21450
F	-1.77380	-3.64710	-3.57820	H	-7.16510	-3.46570	-1.01980	H	5.83740	1.41150	5.14820
F	-2.09620	-1.88210	-4.76690	N	-6.87950	-0.44230	-0.15060	N	4.87090	0.70220	3.43410
C	-0.63620	2.14550	4.41880	N	-6.48900	-2.45800	0.68530	N	4.53010	2.72650	2.60540
H	0.07120	2.38720	5.21150	C	-6.72300	-1.14780	1.01420	C	4.63170	1.40080	2.28030
H	-1.23550	1.30450	4.76970	O	-3.51080	-1.92400	-2.48360	O	4.60310	0.93660	1.16330
H	-1.28870	3.00420	4.27370	O	-6.85270	-0.70910	2.13690	O	1.99490	2.58830	6.26970
N	3.36920	4.46860	1.40170	N	-4.02470	3.22720	-2.45890	C	2.95750	4.74070	4.64820
C	2.90250	5.53960	2.24390	C	-5.28230	3.54800	-1.83820	H	3.80780	5.42170	4.78290
C	1.75160	6.16820	1.41680	C	-6.08470	2.22510	-1.94560	H	2.24780	4.83300	5.47870
N	1.74170	5.38180	0.21300	N	-5.15750	1.33450	-2.59370	C	4.60080	3.76260	1.61440
C	2.67050	4.37620	0.23040	C	-3.94480	1.91500	-2.84330	H	4.84810	3.27310	0.66540
H	3.73140	6.21880	2.48820	H	-5.74090	4.41810	-2.32760	H	5.37890	4.47980	1.90370
H	1.87240	7.23370	1.17590	H	-7.01870	2.28440	-2.52070	C	3.69470	0.30240	6.46460
N	2.24780	5.10750	3.45410	N	-5.19090	3.76780	-0.41360	H	4.67570	0.17590	6.93970
N	0.59470	5.97970	2.25950	N	-6.37160	1.89350	-0.57070	H	2.94130	0.61190	7.19820
C	0.90780	5.39310	3.45900	C	-5.84810	2.80510	0.30890	C	5.33370	-0.65600	3.41940
O	2.88820	3.59980	-0.67310	O	-2.99880	1.39310	-3.38930	H	6.24650	-0.73060	4.02280
O	0.14800	5.23580	4.38970	O	-6.00550	2.80570	1.51160	H	5.53330	-0.90690	2.37130
N	3.25440	-3.52050	3.86620	C	-5.50610	0.04210	-3.10570	H	-2.23170	-0.84190	-0.77560
C	3.34640	-3.21730	5.26910	H	-6.47380	0.10600	-3.61620	C	-2.19260	-0.55710	0.27790
C	4.16160	-1.89800	5.29600	H	-4.70930	-0.24110	-3.80330	C	-3.34030	0.76920	2.07660
N	4.38120	-1.61710	3.90090	C	-7.35090	0.91640	-0.17540	C	-2.20410	-1.40730	2.62860
C	3.78430	-2.53440	3.07820	H	-8.21000	0.98150	-0.85430	C	-3.35790	-0.46820	2.96840
H	3.78900	-4.05950	5.81690	H	-7.64300	1.15990	0.85280	C	-2.25680	-1.80180	1.15700
H	5.11700	-1.94230	5.83500	C	-6.53530	-3.52030	1.65210	C	-3.35230	0.37880	0.60200
N	2.08450	-2.84050	5.86400	H	-6.85560	-3.06790	2.59790	H	-2.44240	1.35180	2.28830
N	3.26870	-0.96600	5.93930	H	-7.25640	-4.27630	1.31530	H	-1.25710	-0.90230	2.83830
C	2.05320	-1.52260	6.24180	C	-3.07810	4.20770	-2.90830	H	-4.31010	-0.99230	2.85140
O	3.78430	-2.51100	1.86680	H	-3.61850	5.02860	-3.39600	H	-3.19450	-2.32860	0.96300
O	1.13680	-0.96600	6.80880	H	-2.41430	3.69880	-3.61590	H	-4.29730	-0.12030	0.37580
N	-0.39910	-5.84380	2.45610	C	-4.80890	5.02760	0.16620	H	-1.25420	-0.03220	0.46740
C	-0.60210	-6.00610	3.87060	H	-5.13540	5.00110	1.21220	H	-4.20580	1.39490	2.30830
C	0.72100	-5.47620	4.48170	H	-5.31240	5.83790	-0.37870	H	-2.26070	-2.28920	3.27210
N	1.47760	-5.06700	3.32770	C	-1.23280	-6.43780	1.45160	H	-3.29270	-0.17120	4.01820
C	0.78290	-5.22110	2.15670	H	-1.47880	-7.46230	1.75410	H	-1.43430	-2.47850	0.91070
H	-0.84750	-7.05040	4.10500	H	-0.65560	-6.42810	0.52020	H	-3.28880	1.27720	-0.01850
H	1.29040	-6.20160	5.07800	C	-3.01150	-5.40650	4.38840				
N	-1.60210	-5.12360	4.42470	H	-3.47600	-4.80760	5.18040				
N	0.29040	-4.37640	5.31140	H	-3.16100	-6.47890	4.56920				
C	-1.06440	-4.17410	5.25540	C	-4.58930	-4.30640	-1.28610				
O	1.18020	-4.93480	1.04970	H	-5.38610	-5.00900	-1.56220				

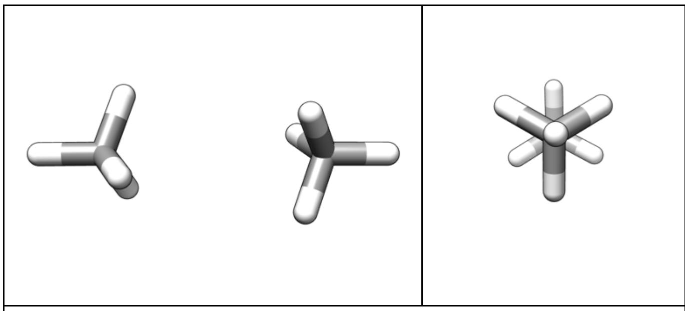	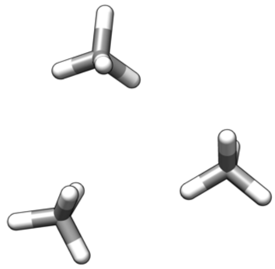
Putative methane dimer (side and top views)	Putative methane trimer

H	-1.04417	1.85784	-0.96463
C	-1.17746	2.12509	0.08615
H	-0.19886	2.22065	0.56243
H	-1.75100	1.34271	0.58874
H	-1.71338	3.07465	0.15727
H	-0.23618	-1.32298	-0.72862
C	0.74879	-1.21063	-0.26943
H	1.30743	-0.43337	-0.79593
H	1.29151	-2.15698	-0.33132
H	0.63022	-0.92449	0.77824

C	-0.70926	2.19231	-0.00015
H	-0.59873	3.27367	-0.11189
H	-1.70209	1.88960	-0.34093
H	0.05053	1.68302	-0.59706
C	2.37323	-0.51861	0.00011
H	2.57064	-0.00650	-0.94498
H	1.43985	-1.08002	-0.07808
H	3.19521	-1.20389	0.22099
H	-0.58652	1.91960	1.05085
H	2.28670	0.21884	0.80139
H	-1.38379	-1.10051	0.88704
C	-1.66405	-1.67388	0.00005
H	-2.74652	-1.82219	-0.01151
H	-1.16327	-2.64507	0.01928
H	-1.36151	-1.12546	-0.89515

8. References

1 F. Diederich, P. J. Stang and R. R. Tykwinski, in *Modern supramolecular chemistry*, Wiley-VCH Verlag GmbH & Co. KGaA, 2008.

2 K. I. Assaf, M. Florea, J. Antony, N. M. Henriksen, J. Yin, A. Hansen, Z. W. Qu, R. Sure, D. Klapstein, M. K. Gilson, S. Grimme and W. M. Nau, *J. Phys. Chem. B*, 2017, **121**, 11144–11162.

3 Gaussian 16, Revision C.01, M. J. Frisch, G. W. Trucks, H. B. Schlegel, G. E. Scuseria, M. A. Robb, J. R. Cheeseman, G. Scalmani, V. Barone, G. A. Petersson, H. Nakatsuji, X. Li, M. Caricato, A. V. Marenich, J. Bloino, B. G. Janesko, R. Gomperts, B. Mennucci, H. P. Hratchian, J. V. Ortiz, A. F. Izmaylov, J. L. Sonnenberg, D. Williams-Young, F. Ding, F. Lipparini, F. Egidi, J. Goings, B. Peng, A. Petrone, T. Henderson, D. Ranasinghe, V. G. Zakrzewski, J. Gao, N. Rega, G. Zheng, W. Liang, M. Hada, M. Ehara, K. Toyota, R. Fukuda, J. Hasegawa, M. Ishida, T. Nakajima, Y. Honda, O. Kitao, H. Nakai, T. Vreven, K. Throssell, J. A. Montgomery, Jr., J. E. Peralta, F. Ogliaro, M. J. Bearpark, J. J. Heyd, E. N. Brothers, K. N. Kudin, V. N. Staroverov, T. A. Keith, R. Kobayashi, J. Normand, K. Raghavachari, A. P. Rendell, J. C. Burant, S. S. Iyengar, J. Tomasi, M. Cossi, J. M. Millam, M. Klene, C. Adamo, R. Cammi, J. W. Ochterski, R. L. Martin, K. Morokuma, O. Farkas, J. B. Foresman, and D. J. Fox, Gaussian, Inc., Wallingford CT, 2016.

4 *TURBOMOLE V7.2.1 2015, a development of University of Karlsruhe and Forschungszentrum Karlsruhe GmbH, 1989-2007, TURBOMOLE GmbH, since 2007; available from http://www.turbomole.com*.

5 R. Ahlrichs, M. Bär, M. Häser, H. Horn and C. Kölmel, *Chem. Phys. Lett.*, 1989, **162**, 165–169.

6 A. Schäfer, C. Huber and R. Ahlrichs, *J. Chem. Phys.*, 1994, **100**, 5829–5835.

7 M. Sierka, A. Hogekamp and R. Ahlrichs, *J. Chem. Phys.*, 2003, **118**, 9136–9148.

8 P. Deglmann, K. May, F. Furche and R. Ahlrichs, *Chem. Phys. Lett.*, 2004, **384**, 103–107.

9 S. Grimme, C. Bannwarth and P. Shushkov, *J. Chem. Theory Comput.*, 2017, **13**, 1989–2009.

10 S. Grimme, *J. Chem. Theory Comput.*, 2019, **15**, 2847–2862.
11 C. Bannwarth, S. Ehlert and S. Grimme, *J. Chem. Theory Comput.*, 2019, **15**, 1652–1671.
12 S. Ehlert, M. Stahn, S. Spicher and S. Grimme, *J. Chem. Theory Comput.*, 2021, **17**, 4250–4261.
13 J. Tao, J. P. Perdew, V. N. Staroverov and G. E. Scuseria, *Phys. Rev. Lett.*, 2003, **91**, 146401.
14 F. Weigend and R. Ahlrichs, *Phys. Chem. Chem. Phys.*, 2005, **7**, 3297–3305.
15 F. Weigend, *Phys. Chem. Chem. Phys.*, 2006, **8**, 1057–1065.
16 S. Grimme, J. Antony, S. Ehrlich and H. Krieg, *J. Chem. Phys.*, 2010, **132**, 154104.
17 S. Grimme, S. Ehrlich and L. Goerigk, *J. Comput. Chem.*, 2011, **32**, 1456–1465.
18 I. Funes-Ardoiz and R. S. Paton, *Zenodo*, 2018, DOI: 10.5281/zenodo.1435820.
19 R. F. Ribeiro, A. V. Marenich, C. J. Cramer and D. G. Truhlar, *J. Phys. Chem. B*, 2011, **115**, 14556–14562.
20 S. Grimme, *Chem. - Eur. J.*, 2012, **18**, 9955–9964.
21 Y.-P. Li, J. Gomes, S. M. Sharada, A. T. Bell and M. Head-Gordon, *J. Phys. Chem. C*, 2015, **119**, 1840–1850.
22 A. Hellweg and F. Eckert, *AIChE J.*, 2017, **63**, 3944–3954.
23 J. P. Perdew, *Phys. Rev. B*, 1986, 34, 7406.
24 J. P. Perdew, *Phys. Rev. B*, 1986, **33**, 8822–8824.
25 A. D. Becke, *Phys. Rev. A*, 1988, **38**, 3098–3100.
26 A. Klamt and G. Schüürmann, *J. Chem. Soc. Perkin Trans. 2*, 1993, 799–805.
27 *Eckert F, Klamt A. COSMOtherm, Version C30, Release, 17.01. Leverkusen, Germany: COSMOlogic GmbH & Co. KG, 2016*, .
28 M. Florea and W. M. Nau, *Angew. Chem. Int. Ed.*, 2011, **50**, 9338–9342.
29 K. I. Assaf and W. M. Nau, *Supramol. Chem.*, 2014, **26**, 657–669.
30 X. Lu and L. Isaacs, *Angew. Chem. Int. Ed.*, 2016, **55**, 8076–8080.
31 R. Rabbani and E. Masson, *Org. Lett.*, 2017, **19**, 4303–4306.
32 S. J. Barrow, K. I. Assaf, A. Palma, W. M. Nau and O. A. Scherman, *Chem. Sci.*, 2019, **10**, 10240–10246.
33 R. A. Kendall, T. H. D. Jr. and R. J. Harrison, *J. Chem. Phys.*, 1992, **96**, 6796–6806.
34 M. Ernzerhof and G. E. Scuseria, *J. Chem. Phys.*, 1999, **110**, 5029–5036.
35 C. Adamo and V. Barone, *J. Chem. Phys.*, 1999, **110**, 6158–6170.
36 D. Ben-Amotz and D. R. Herschbach, *J. Phys. Chem.*, 1990, **94**, 1038–1047.
37 T. N. Olney, N. M. Cann, G. Cooper and C. E. Brion, *Chem. Phys.*, 1997, **223**, 59–98.
